# Quantitative Prediction of Stress Relaxation Kinetics in Dissociative Covalent Adaptable Networks

**DOI:** 10.1021/acs.macromol.5c02070

**Published:** 2025-12-03

**Authors:** Jessica Mangialetto, Osman Konuray, Sasan Moradi, Xavier Fernández-Francos, Joost Brancart, Niko Van den Brande, Xavier Ramis, Guy Van Assche

**Affiliations:** † Sustainable Materials Engineering, 70493 Vrije Universiteit Brussel (VUB), Pleinlaan 2, Brussels 1050, Belgium; ‡ Thermodynamics Laboratory, ETSEIB, 16767 Universitat Politècnica de Catalunya, Av. Diagonal 647, Barcelona 08028, Spain

## Abstract

A kinetic-structural model was developed to quantitatively describe the stress relaxation behavior of covalent adaptable networks based on the reversible Diels–Alder chemistry. The model draws on the analogy between stress relaxation and network de-cross-linking, where residual stress is attributed to elastically active network strands. A recursive network analysis, based on the Macosko–Miller approach, is coupled with a model that incorporates the reversible Diels–Alder reaction kinetics, stress-induced bond activation, and the reduced efficiency of bond exchanges during late-stage relaxation. The model was validated using rheological data from two Diels–Alder-based networks, with cross-linking kinetics and equilibrium conversions used to predict initial stresses. The stress relaxation behavior, including characteristic times and shape parameters across varying temperatures and cross-link densities, was accurately predicted using only two temperature-independent parameters. Beyond predictive capabilities, the model enables the extraction of kinetic and thermodynamic parameters from experimental data, supporting its use in both direct simulation and inverse design. Thanks to its low computational cost, the framework facilitates rapid exploration of compositional and structural scenarios, aiding the design of application-specific materials. This approach offers a robust and efficient tool for bridging the gap between dynamic covalent chemistries and the development of functional materials and their advanced processing.

## Graphical abstract

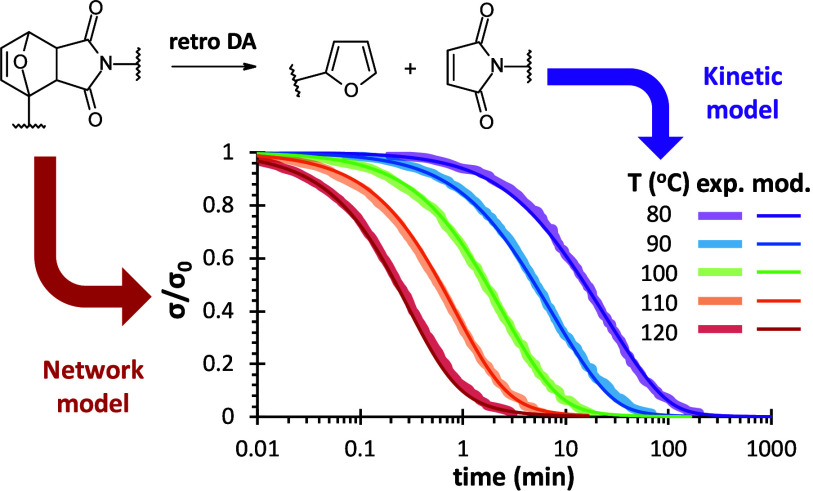

## 1 Introduction

Dynamic covalent polymer networks are polymer networks that are chemically cross-linked by dynamic covalent chemistries, and are often referred to as covalent adaptable networks (CAN).^1–5^ They combine the superior mechanical properties and stability of covalently cross-linked polymer networks with the (re)­processability of thermoplastics when activated using the adequate stimulus, typically heat or light irradiation.^6^ Successful material design and exploitation of the (re)­processability of CANs require a detailed knowledge and understanding of the dynamic chemical reactions taking place and their impact on the thermal, viscoelastic, and rheological properties of the material in different reprocessing scenarios.

A wide range of methodologies can be used and combined to study chemical exchange reactions and the structure and properties of CANs.^7–10^ In particular, stress relaxation analysis can provide valuable insights in the dynamic nature of the dynamic covalent chemistries and can reveal interesting features, such as the existence of simple or complex relaxation behavior when combining dynamic bonds with different kinetics,^10–14^ incomplete relaxation due to the presence of permanent bonds within the network structure,^15,16^ or structural effects on the kinetics of the dynamic bond exchange reactions.^17–19^ Moreover, stress relaxation experiments provide valuable information about the application and (re)­processing conditions and on the limitations of these materials.^10^ The stress relaxation capability constitutes the basis for advanced functionalities of dynamic covalent polymer networks, such as self-healing,^20–22^ reaction-induced shape-memory response,^10,23^ and advanced (re)­processability.^24^

Thermoreversible dissociative covalent polymer networks undergo a reversible network depolymerization (dissociation) upon heating, due to the temperature-dependent equilibrium between the forward and reverse reactions. This results in a decrease in cross-link density with increasing temperature, and eventually, in most cases, in degelation of the polymer network, yielding a viscous liquid.^16,25–27^ This is in clear contrast with associative covalent polymer networks, which show no change in cross-link density upon activation of the dynamic covalent chemistries: increasing the intensity of the stimulus (e.g., a higher temperature) results in an acceleration of the dynamic exchange reactions, without a net change in network connectivity. In some cases, the reaction equilibrium of dissociative covalent chemistries is hardly disturbed by temperature,^26^ or temperature conditions can be found where the effect is minimal.^16^ In any case, it is possible to observe the stress relaxation processes, provided the temperature is constant and below the gel transition temperature and the dynamic bond exchange is at equilibrium, so that the material retains structural integrity and steady state conditions (constant cross-link density).^14,16,25,26,28^

The Diels–Alder (DA) reaction is a concerted thermoreversible [4+2] cycloaddition reaction and is among the most extensively studied dissociative dynamic covalent chemistries.^29^ A wide range of diene-dienophile pairs has been reported for DA reactions, with the furan-maleimide system being the most studied for self-healing applications and dissociative covalent networks.^20,21,30,31^ The Diels–Alder reaction kinetics have been studied elaborately in recent years.^8,30,32,33^ The cycloaddition of substituted furan and maleimide groups leads to the formation of *endo* and *exo* stereoisomers with distinct reaction kinetics and thermodynamics (Scheme 1).^8,33^ The DA adducts form at temperatures around room temperature, while the retro DA reactions start to become important at relatively low temperatures from ∼75 °C, making this chemistry suitable for CANs. The DA reaction is particularly valued for its versatility in creating materials with diverse architectures and properties, along with its orthogonality to numerous functional groups.^34^ A consistent set of kinetic parameters involving the formation of *exo* and *endo* Diels–Alder adducts has been determined and can be used to describe the cross-linking of a wide range of DA networks based on the combination of furan and maleimide components with polyether backbone and different functionalities.^33^ It is worth noting that this set of kinetic parameters is valid throughout the entire conversion range of the cross-linking process of these polyether-based DA networks, irrespective of the state of cross-linking.^32,33^ The forward and reverse reactions are not affected by the limitations of chain diffusion and mobility in the network structure, other than those corresponding to the effect of vitrification on the global segmental mobility of the material depending on the processing temperature.^9^ The use of the maleimide chemistry at elevated temperatures is limited by side reactions. The most common side reactions are the Michael addition between amines and maleimides above 100 °C^35^ and the maleimide homopolymerization above 150 °C.^35–37^ These reactions consume maleimide groups to create irreversible bonds, reducing the system’s thermal reversibility, which was also shown to be potentially useful to tune the polymer properties by controlled thermal annealing.^38^

**Scheme 1:**
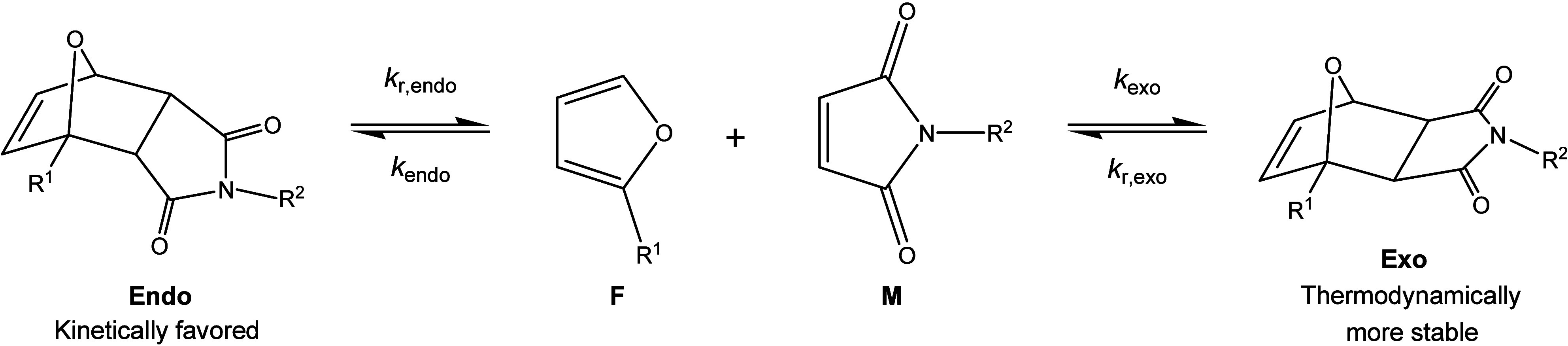
Formation of *endo* and *exo* Adducts in the Furan-Maleimide Diels–Alder Reaction, with *k*_endo_ and *k*_exo_, the Rate Constants for the Diels–Alder Reactions, and *k*_r,*endo*_ and *k*_r,*exo*_ Those of the Retro Diels–Alder Reaction

The thermoreversible DA adducts can be broken in a reversible fashion by mechanical force, resulting in the conception of chemically cross-linked polymer networks capable of recovering their structure and properties after damage healing, either thermally mediated and under ambient conditions.^20^ The thermally dynamic and reversible nature of these networks also enables reshaping, reprocessing, and recycling, which are often unattainable in conventional cross-linked polymers. DA-based networks have found a wide range of applications, significantly extending the lifetime of coatings,^39,40^ composites,^41,42^ photovoltaic panels,^43–45^ soft robotics,^24,46–48^ electronics,^49^ biomedical materials,^50,51^ and many others.^22^ It has also sparked interest in extrusion-based additive manufacturing.^10,24,52^

As their relaxation behavior plays a crucial role in applications and processing, from a practical perspective, it would be useful to define some criteria that facilitate formulation design to tailor the bond exchange-driven relaxation behavior. Although it is claimed that the stress relaxation of CANs can be represented using simple Maxwell relaxation models,^53–55^ it is acknowledged that stretched exponential (i.e., Kolsrauch–Williams–Watts) functions provide a better description of the process considering the existence of a certain distribution of relaxation times.^7,15,17,56,57^ In contrast, other researchers have proposed the use of multiple Maxwell elements to model broad or discrete distributions of relaxation times, which would be caused by the coexistence of different relaxation processes with different relaxation kinetics.^7,14,58^ The latter approach certainly improves the mathematical fitting of experimental data in comparison with simple models, with increased mechanistic modeling value, albeit at the risk of a loss of significance of these parameters when too many are defined. It can be difficult to relate the parameters of these models to the chemical structure or topological features of the CANs, or to the bond exchange events taking place, therefore limiting their predictive capabilities.

Significant efforts have been made to model and to simulate the effect of bond exchange on the properties of different dynamic systems, such as transient polymer networks and CANs.^53,55,59–69^ These efforts provide valuable insights into the mechanical, viscoelastic, and rheological properties of the material in various scenarios, and into the reprocessing capabilities that can be attained. However, it is difficult to translate the outcomes of these simulations into real scenarios for many reasons, namely: (i) many studies overlook the wide range of network topologies present in these materials and their effect on bond exchange dynamics, (ii) detailed computational simulations are expensive and time-consuming, (iii) certain assumptions can be easily challenged, such as the Maxwell-type exponential decay of elastically active cross-links, and (iv) permanent network effects and complex bond exchange kinetics are often neglected or addressed in a nonspecific manner.

It was recently proposed that the stress relaxation process in CANs could be likened to a network de-cross-linking process, in which the exchange of bonds leads to a deactivation of the elastic activity of the network strands, which is similar to the cleavage of these dynamic bonds,^70^ as illustrated in Figure 1. In the bond exchange process, some nonexchanged bonds would lose elastic activity and therefore be deactivated. Based on this analogy, a recursive network analysis methodology was developed,^70^ inspired by the recursive network build-up methods of Macosko and Miller^71^ and by that of Williams and co-workers.^72^ Making use of this model, a number of expressions were derived, describing (i) the evolution of the network structure and the remaining stress, (ii) the extent of bond exchange required for complete relaxation of stress, and (iii) the upper threshold for the fraction of permanent bonds allowing complete stress relaxation. This enabled the development of relaxation maps to identify viable reprocessing scenarios.^70^ Coupling the structural model with a kinetic model describing the rate of bond exchange, a number of kinetic trends depending on the topology and composition of the network structure, as well as the existence of diverse bond exchange processes, could be identified and rationalized.^70^ Indeed, a number of experimental results reported in the literature could be rationalized on the basis of this network structural-kinetic coupling approach.^70^ Konuray and co-workers used this methodology in the modeling of various CANs based on thiol–ene disulfide networks,^73,74^ dual-curing and dual-relaxing thiol-isocyanate-epoxy networks,^13^ and epoxy-amine networks based on aromatic disulfide structures.^75^ The results suggested the possibility of optimizing the formulation composition and the dynamic component content, while ensuring complete stress relaxation and therefore full reprocessing capabilities.

**Figure 1:**
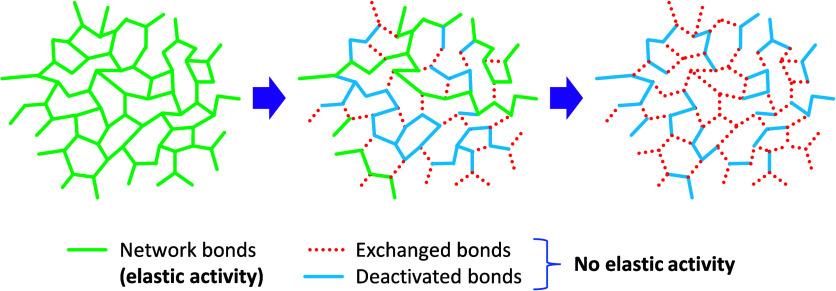
Representation of the effect of the bond exchange process by analogy with a de-cross-linking process.

It was also proposed that quantitative predictions of stress relaxation processes for dissociative networks can be made, provided that detailed kinetic data were available.^70^ The goal of this paper is to show that our kinetic-structural modeling approach can be used for the quantitative prediction of stress relaxation kinetics of Diels–Alder reversible networks. First, the modeling approach will be validated against a benchmark analytical model that describes the deactivation of the elastic activity of the network structure during a stress relaxation process. Some of the kinetic model parameters will be redefined to provide a more accurate representation of the phenomenon. The model will first be used to simulate the network formation kinetics, considering the temperature-dependent equilibrium network structure and cross-link density. Second, the kinetic-structural model will be used to calculate stress relaxation curves at different temperatures, taking as a starting point these equilibrium network structures. We will use detailed kinetic information about the furan-maleimide Diels–Alder reversible reaction available in the literature,^8,33^ to simulate the cross-linking and relaxation of a certain set of polymer networks obtained from monomers with various functionalities. The cross-linking and stress relaxation processes for these polymer networks will be analyzed with rheological experiments under different temperature conditions. A number of model outputs will be compared with experimental data: gelation and cross-linking kinetics, equilibrium network structure, relaxation time, and activation energy. The validity of the hypotheses underlying the analysis of these reversible Diels–Alder CANs will be examined in detail. The general validity of this modeling framework for other associative/dissociative CANs will be discussed and future research directions will be proposed.

## 2 Theoretical

### 2.1 Diels–Alder Reaction Kinetics

The Diels–Alder reaction kinetics of furan-bismaleimide networks are determined according to the kinetic model reported in the literature, taking into consideration the formation of *exo* and *endo* adducts having different kinetics and thermodynamic stability.^8,33^ If we call A the furan group, B the maleimide group, and AB the furan-maleimide adduct, we can write for the *endo* and *exo* adducts:

$$A+B⇄{\mathrm{AB}}_{endo}A+B⇄{\mathrm{AB}}_{exo}$$ 1

By considering the concentration of the different *i* species, [*i*] in mol kg^–1^, we can write the following rate expressions for the concentration of *endo* and *exo* adducts:

$$\begin{array}{c} \frac{d{\left[\mathrm{AB}\right]}_{endo}}{dt}=k_{\mathrm{DA},endo}\cdot [A]\cdot [B]-k_{\mathrm{rDA},endo}\cdot {\left[\mathrm{AB}\right]}_{endo}\\ \frac{d{\left[\mathrm{AB}\right]}_{exo}}{dt}=k_{\mathrm{DA},exo}\cdot [A]\cdot [B]-k_{\mathrm{rDA},exo}\cdot {\left[\mathrm{AB}\right]}_{exo} \end{array}$$ 2

where *k*_DA,*endo*_ and *k*_DA,*exo*_ are the rate constants of the forward Diels–Alder reaction leading to the *endo* and *exo* adducts, respectively, and *k*_rDA,*endo*_ and *k*_rDA,*exo*_ are the rate constants of the retro Diels–Alder reaction of these same adducts. The different rate constants have Arrhenius temperature dependence:

$$k_{i}=k_{0,i}\cdot \exp\left(-E_{i}/R\cdot T\right)$$ 3

The kinetic parameters of the different rate constants are reported in the literature^33^ and are also shown in Table S1 in the Supporting Information.

In a stoichiometric system, the concentrations of furan and maleimide relate as follows:

$${\left[A\right]}_{0}={\left[B\right]}_{0}\;\;[A]={\left[A\right]}_{0}\cdot (1-x)=[B]$$ 4

where *x* is the global conversion of the reaction, which is equal to the contribution of the *endo* and *exo* adducts as *x* = *x*_*endo*_ + *x*_*exo*_. The concentration of Diels–Alder adducts can then be expressed as

$$\begin{array}{c} {}[\mathrm{AB}]={\left[A\right]}_{0}\cdot x\\ {}[\mathrm{AB}]={\left[\mathrm{AB}\right]}_{endo}+{\left[\mathrm{AB}\right]}_{exo}={\left[A\right]}_{0}\cdot x_{endo}+{\left[A\right]}_{0}\cdot x_{exo} \end{array}$$ 5

Rate expressions depending on the extent of conversion can easily be derived. A temperature-dependent equilibrium conversion *x*_eq_ can also be obtained in a straightforward fashion by simply setting the sum of the forward and reverse reaction rates equal to zero for each stereoisomer.

$$x_{\mathrm{eq}}=\frac{(1+2\cdot {K^{\prime }}_{\mathrm{eq}})-\sqrt{1+4\cdot {K^{\prime }}_{\mathrm{eq}}}}{2\cdot {K^{\prime }}_{\mathrm{eq}}}$$ 6

where ${K^{\prime }}_{\mathrm{eq}}$ is a dimensionless global equilibrium constant determined from the contribution of the different forward and reverse reactions. Details of the complete equilibrium and kinetic model are given in SI.

### 2.2 Network Build-Up Model

The reaction of A and B species leads to an AB adduct. This can be represented as the formation of a reacted species containing (+) and (−) bonds that are connected with each other, as seen in Scheme 2. According to this definition, a reacting system can be represented as a population of fragments containing a number of (+) or (−) bonds, depending on the state of reaction, that are randomly connected with each other, leading to a probable polymer distribution or network structure. Based on these assumptions, a network build-up model based on the recursive approach of Macosko–Miller^71^ (M–M, hereafter) and that of Williams^72^ and co-workers is used.

**Scheme 2:**
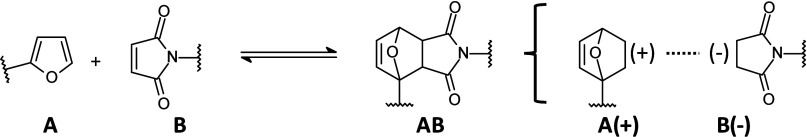
Diels–Alder Adduct Formation and Separation into Structural Fragments with (+) and (−) Bonds

Let us consider the furan- and maleimide-containing species, *A*_f_ and *B*_g_ with functionalities *f* and *g*, respectively. In stoichiometric conditions, the initial concentration of these species must fulfill the following mass balance.

$${\left[A_{f}\right]}_{0}\cdot f={\left[B_{g}\right]}_{0}\cdot g\Leftrightarrow {\left[A\right]}_{0}={\left[B\right]}_{0}$$ 7

For a given global extent of DA conversion *x*, a distribution of structural fragments *A*_f,n_ and *B*_g,n_ can be calculated. *A*_f,n_ represents the furan structural fragments with *n* reacted (+) bonds, and *B*_g,n_ represents the maleimide structural fragments with *n* reacted (−) bonds. The total number of (+) bonds equals the total number of (−) bonds at all times. Fragments with (+) and (−) bonds are randomly combined with each other leading to a probable polymer or network structure.

Pregel statistical averages are calculated from the definition of expected weights *W* ^+^ and *W* ^–^ pending from (+) and (−) bonds, respectively. Gelation is determined from the condition that these *W* ^+^ and *W* ^–^, and therefore the mass-average molecular weight *M*_w_, tend to infinity, which leads to the well-known expression for gel point conversion *x*_gel_ for a stoichiometric system:

$$x_{\mathrm{gel}}=\sqrt{\frac{1}{\left(f-1)\cdot (g-1\right)}}$$ 8

Postgel statistics are determined from the definition of the extinction probabilities *Z* ^+^ and *Z* ^–^ that represent the likelihood of finding a finite branch when looking outward from a given bond. The extinction probabilities are used to determine a number of statistical averages, such as the number of elastically active network strands (EANS), *n*_EANS_, which will contribute to the elastic mechanical response of the network structure, given by the well-known expression for the rubbery modulus:

$$G=\phi \cdot R\cdot T\cdot (n_{\mathrm{EANS}}\cdot \rho )$$ 9

where *n*_EANS_ is expressed in mol·kg^–1^, ρ is the density in kg·m^–3^, *T* is the absolute temperature in K, *R* is the gas constant in J·mol^–1^·K^–1^ and ϕ is the so-called front factor, which depends on the distribution of end-to-end distances of network strands, chain stiffness and steric restrictions, among other factors.^72,76^

Detailed information on the recursive methodology and a complete derivation of all structural averages in the pregel and postgel stages is given in SI.

### 2.3 Stress Relaxation Kinetics

Let us consider the bond exchange process leading to the relaxation of stress. At the beginning of the process, we can label all the dynamic bonds D as nonexchanged bonds, having a concentration [D]_n–e_. As the bond exchange advances, these dynamic bonds are transformed into exchanged bonds, with a concentration [D]_e_. These exchanged bonds can continue participating in bond exchange events, but their status of exchanged bonds is not affected:

$$\begin{array}{c} {\left[D\right]}_{n-e}\overset{\mathrm{exchange}}{\to }{\left[D\right]}_{e}\\ {\left[D\right]}_{e}\overset{\mathrm{exchange}}{\to }{\left[D\right]}_{e} \end{array}$$ 10

From a kinetics perspective, this can be expressed as

$$\frac{d{\left[D\right]}_{n-e}}{dt}=-k_{\mathrm{xch}}\cdot {\left[D\right]}_{n-e}\;\;\frac{d{\left[D\right]}_{e}}{dt}=k_{\mathrm{xch}}\cdot {\left[D\right]}_{n-e}$$ 11

where *k*_xch_ is the temperature-dependent apparent bond exchange rate constant. Depending on the type of bond exchange process, this rate constant may depend on the total concentration of dynamic bonds and/or catalytic species, the reactivity of the dynamic bonds or the mobility of the network structure, among other factors.

The total number of bonds must remain constant:

$$\begin{array}{c} \frac{d{\left[D\right]}_{n-e}}{dt}+\frac{d{\left[D\right]}_{e}}{dt}=0\\ {\left[D\right]}_{n-e}+{\left[D\right]}_{e}={\left[D\right]}_{\mathrm{total}}=\mathrm{ct} \end{array}$$ 12

Under isothermal conditions, the equilibrium between forward and reverse reactions in dissociative CANs ensures the total number of bonds remains constant in a stress relaxation process, providing the relaxation time constant of the reaction equilibrium is fast with respect to the time scale of the mechanical relaxation.

Following the previous work of Konuray et al.,^70^ the bond exchange process leading to the deactivation of the elastic activity of the network strands produces a similar effect to that of bond cleavage in a network de-cross-linking process. Following this analogy, complete stress relaxation would occur when “degelation” is reached in this de-cross-linking-like process. A recursive network analysis methodology based on the Macosko–Miller method, coupled to a kinetic model describing bond exchange/cleavage kinetics, is used to describe stress relaxation kinetics and network structure effects. In this work, an additional analytical model is defined that considers (i) the deactivation of a network strand in the stress relaxation process, and (ii) that only bond exchange events within the network structure produce an impact on the stress relaxation. This model is used to discuss the validity of the above hypotheses and the outputs produced by the general recursive model.

In dissociative networks, the kinetics of the bond exchange process are related to the kinetics of the bond-breaking reaction.^25,70^ While the forward reactions do take place, the newly formed bonds lead to the reconstruction of network chains without elastic activity, and in consequence, they can be ignored in the analysis of the stress relaxation process. The recursive method used for modeling stress relaxation is adapted to incorporate the temperature-dependent network structure equilibrium and the coexistence of reverse reactions with different kinetics, leading to a complex relaxation behavior depending on the contribution of *endo* and *exo* adducts in the equilibrium network structure.

#### Analytical Model

In this analytical model, it is assumed that during stress relaxation a bond exchange event on a given network strand deactivates the whole strand. Therefore, only bond exchange events that take place in nondeactivated network strands produce an effect. The general process of deactivating network strands is illustrated in Figure 2a and illustrated locally in Figure 2b. In addition to deactivating the network strand, a bond exchange will decrease the functionality of the cross-linking units at strand ends, eventually producing unbranched active units belonging to an active chain.

**Figure 2:**
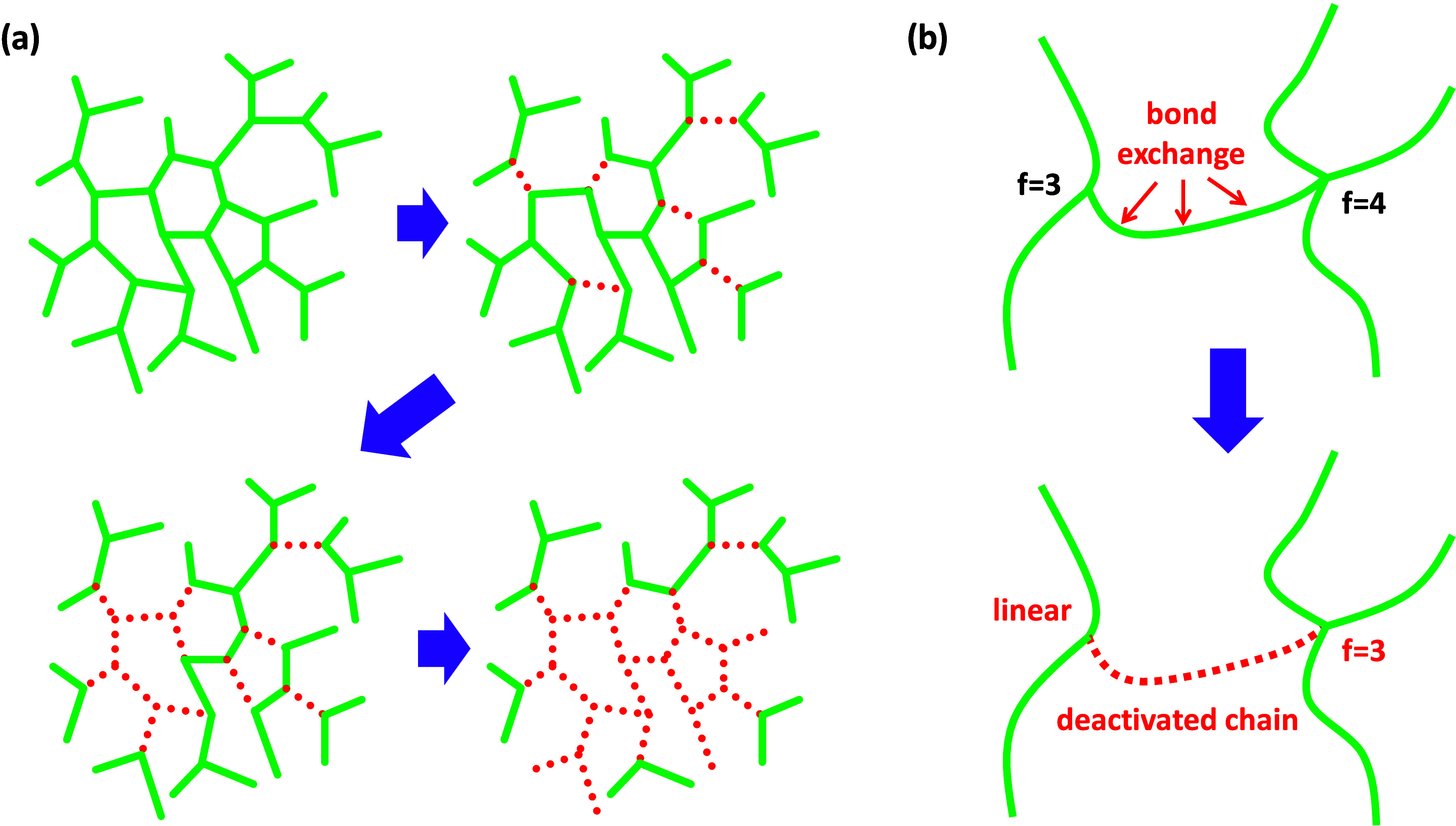
Representation of the analytical model for stress relaxation, showing (a) process of transforming network strands (green, solid) into deactivated network strands (red, dotted) and (b) detail of the effect of a bond exchange event in one network strand.

A kinetic model can be defined taking into consideration only the elements that constitute the remaining active network structure. For that purpose, it is considered that, when a network strand is deactivated, all of their constituents are removed from the system and the functionality of the original chain ends are downgraded. In order to avoid confusions with the general network build-up and relaxation models based on the M–M method, in the analytical model we will consider a simplified notation for the different components in the network structure. Components will be identified as *A*_f,net_ and *B*_g,net_, where f and g are the effective functionality of the component in the active network structure (net). Therefore, for a fully cross-linked A_4_–B_2_ network, the relaxation process starts from *A*_4,net_ and *B*_2,net_ components. In the bond exchange and chain deactivation process, the *A*_4,net_ elements are successively downgraded to *A*_3,net_ and *A*_2,net_ elements, as illustrated in Figure 2b; *A*_2,net_ and *B*_2,net_ elements from the network strand affected by the bond exchange are deactivated and therefore removed from the system.

*A*_f,net_ components contain (+)_net_ bonds that are connected to *B*_2,net_ components contain (−)_net_ bonds. The kinetic model must ensure that the number of (+)_net_ and (−)_net_ bonds in the active network, *n*_(+),net_ and *n*_(−),net_ respectively, must be equal throughout the process.

$$n_{(+),\mathrm{net}}=4\cdot [A_{4,\mathrm{net}}]+3\cdot [A_{3,\mathrm{net}}]+2\cdot [A_{2,\mathrm{net}}]=2\cdot [B_{2,\mathrm{net}}]=n_{(-),\mathrm{net}}$$ 13

Given that all the deactivated elements are removed from the system, all the remaining components contribute to the active network structure (neither any pending chains nor any soluble fraction are present). Any further bond exchange is only effective on this remaining active network structure. The rate of bond exchange *r*_xch_ depends on the concentration of remaining dynamic groups, given by (+)_net_ or (−)_net_ bonds, as follows:

$$r_{\mathrm{xch}}=k_{\mathrm{xch}}\cdot n_{(+),\mathrm{net}}=k_{\mathrm{xch}}\cdot n_{(-),\mathrm{net}}=k_{\mathrm{xch}}\cdot 2\cdot [B_{2,\mathrm{net}}]$$ 14

where *k*_xch_ is the bond exchange rate constant. A detailed kinetic model for the change in the different species in the network structure is found in the Supporting Information.

The fraction of bond exchange events that have taken place is calculated as

$$f_{\mathrm{xch}}=\frac{\int r_{\mathrm{xch}}\cdot dt}{2\cdot {\left[B_{2,\mathrm{net}}\right]}_{0}}=\frac{\int (k_{\mathrm{xch}}\cdot 2\cdot [B_{2,\mathrm{net}}])\cdot dt}{2\cdot {\left[B_{2,\mathrm{net}}\right]}_{0}}$$ 15

Given that all the components in the system contribute to the network structure, the total number of EANS, *n*_EANS_, is simply calculated from the remaining *A*_4,net_ and *A*_3,net_ units in the network structure and by assuming a phantom network model as

$$n_{\mathrm{EANS}}=\left(\frac{4}{2}-1\right)\cdot [A_{4,\mathrm{net}}]+\left(\frac{3}{2}-1\right)\cdot [A_{3,\mathrm{net}}]$$ 16

The fractional stress during the relaxation process is finally calculated from the remaining EANS as

$$f_{\mathrm{stress}}=\frac{\sigma }{\sigma_{0}}=\frac{n_{\mathrm{EANS}}}{n_{\mathrm{EANS},t=0}}$$ 17

where *n*_EANS,*t*=0_ is the number of EANS at the beginning of the stress relaxation process, σ is the stress, σ_0_ is the initial stress. This model is described in detail in the Supporting Information.

#### Macosko–Miller (M–M) Stress Relaxation Model

As was mentioned earlier, the stress relaxation process is studied by analogy to a network de-cross-linking process.^70^ It is hypothesized that the random exchange of bonds leads to a network-wide distribution of bond states that are characterized as either deactivated or nondeactivated. This is similar to bond cleavage during de-cross-linking, which would lead to the formation of pending chains and soluble fractions. Following this analogy, complete stress relaxation is attained when “degelation” occurs in this de-cross-linking-like process.^70^ This process is illustrated in Figure 1, clearly showing the presence of deactivated nonexchanged bonds in the course of the stress relaxation process and at the end.

The general rate expression for the stress relaxation process is as follows:^70^

$$\frac{dx_{\mathrm{xch}}}{dt}=f_{\mathrm{eff}}\cdot k_{\mathrm{xch}}\cdot (1-x_{\mathrm{xch}})$$ 18

where *x*_xch_ is the extent of bond cleavage/exchange, *k*_xch_ is the bond exchange rate constant, and *f*_eff_ is an effectiveness factor. This is equivalent to considering the following expression,

$$\frac{dx}{dt}=-f_{\mathrm{eff}}\cdot k_{\mathrm{xch}}\cdot x$$ 19

In which we use the fraction of existing bonds *x*, that is equivalent to 1 – *x*_xch_. The negative sign indicates that the number of bonds connecting fragments decreases in the course of the stress relaxation process. This is the expression that will be used in this work.

Based on *x*, a population of structural fragments *A*_f,n_ and *B*_g,n_ with a different number of bonds (+) and (−) bonds is generated and used in the recursive procedure. Using the concepts of expected weight and extinction probability, a number of expressions can be derived describing (i) the evolution of the network structure and the remaining stress, and (ii) the extent of bond exchange required for complete relaxation of stress. In the present case no permanent bonds are considered: all the bonds connecting structural fragments can be exchanged. In the case of a fully cross-linked network, the relaxation process would start with *x* = 1, i.e., a fully connected network, and would end when “degelation” is reached, *x* = *x*_gel_. However, in the case of Diels–Alder networks, the relaxation process may start from the initial equilibrium network structure, with *x* = *x*_eq_. This implies that the extent of bond exchange required to relax the network depends on the temperature-dependent equilibrium conversion of the Diels–Alder network.

The rate equation is integrated using discrete timesteps Δ*t* ensuring numerical stability and accuracy. At each integration step, the network structure is evaluated making use of the same recursive procedures described in Section 2.2. Given that the extent of connection *x* is in fact equivalent to the conversion in a cross-linking process, the same expressions used for the evaluation of a network build-up process can be used for the analysis of the stress relaxation process. In particular, it is of interest to calculate (i) *n*_EANS_ (see Supporting Information for details), which is used to determine the fractional stress *f*_stress_ using eq 17, and (ii) the effectiveness factor *f*_eff_, which is critical for the correct determination of the relaxation kinetics. In our previous work, an expression analogous to this was employed:

$$f_{\mathrm{eff}}=\frac{n_{(+),\mathrm{net}}}{n_{(+),\mathrm{net},t=0}}$$ 20

where *n*_(+),net_ is the number of (+) dynamic bonds belonging to the network structure (see Supporting Information for details on the calculation using the M–M method). This definition of *f*_eff_ accounted for the deactivation of nonexchanged chain segments leading to a loss of effectivity of further bond exchange events. It provided an approximately correct description of the general shape of simple stress relaxation processes, for which the stretching parameter β of the stretched exponential KWW model was smaller than one. However, it failed to explain why the value of β varied drastically when comparing apparently simple relaxing systems.

In the present work a novel definition of *f*_eff_ is made. It is presumed that strain-induced stress alters the energy landscape of the dynamic bonds, making them more prone to bond exchange. Such alteration is assumed to cease once the stress is relaxed. Formally, this effectiveness factor *f*_eff_ is defined as

$$f_{\mathrm{eff}}=\exp\left(\frac{n_{(+),\mathrm{EANS}}}{{\left[A\right]}_{0}}\cdot \frac{b}{R\cdot T}\right)=\exp\left(f_{(+),\mathrm{EANS}}\cdot \frac{b}{R\cdot T}\right)$$ 21

The *b* parameter represents the disturbance of the energetic level of the dynamic bonds under stress, which can be related with the conformational changes taking place along the segments in the EANS and a possible distortion of bond angles and distances in Diels–Alder structures. The effect is to decrease the activation energy of the bond exchange process, increasing the bond exchange rate. This can be related to the mechanochemical effects that are described for transient polymer networks.^61^ Other mechanochemical effects have been reported for the dissociation of Diels–Alder adducts.^77^

It is commonly assumed that stress relaxation kinetics do not depend on the level of strain, as long as it is sufficiently small and is not additionally disturbing the molecular landscape.^7^ Accordingly, it is assumed that this energy disturbance *b* does not depend on the level of applied strain. However, this energetic disturbance would depend on *f*_(+),EANS_, which is the fraction of dynamic bonds contributing to the elastic activity of the network, that is, belonging to the EANS. *f*_(+),EANS_ depends on the ratio *n*_(+),EANS_/[A]_0_ where *n*_(+),EANS_ (or its counterpart *n*_(−),EANS_) is the number of dynamic bonds under stress in the relaxing network, and is normalized with respect to the total number of dynamic bonds that would exist in the fully cross-linked network structure, given by [A]_0_. The factor *f*_(+),EANS_ leads to *f*_eff_ > 1 at the beginning of the relaxation process (a positive nonzero value within the exponential), and decreases along the relaxation process, down to *f*_eff_ = 1 once relaxation is complete (i.e., *n*_EANS_ = *n*_(+),EANS_ = *f*_(+),EANS_ = 0). This represents a fundamental difference with respect to our previous definition of *f*_eff_, whose values changed between 1 and 0, from the beginning to the end of relaxation, respectively. It is believed that the new definition represents the stress relaxation kinetics in a more realistic way, since it establishes a base rate of bond exchange regardless of the stress.

A detailed description of the M–M method and the computational algorithms can be found in the Supporting Information.

#### Diels–Alder Relaxation Kinetics

It is commonly assumed that the stress relaxation kinetics in dissociative networks are governed by the reverse reaction, so that the temperature dependence of the relaxation process is identical to that of the reverse reaction.^25,70^ Indeed, the activation energy determined from stress relaxation processes shows fairly constant values regardless of the initial state of dissociation, cross-linking density and the presence of permanent bonds, even if vertical shift factors are used in order to correct the effect of the temperature-dependent cross-linking.^16,25,26,28,57^ In some dissociative CANs, the existence of a percolated network structure may affect the value of the activation energy of the relaxation process,^78^ but such effect is not present in others.^16^

In addition, it is reported that the cross-linking process of Diels–Alder networks starting from the dissociated A and B components is correctly described using a relatively simple set of forward and reverse rate constants that are valid throughout the full conversion range of the process, irrespective of the state of cross-linking.^32,33^ That is, the forward and reverse reactions are not affected by limitations of chain diffusion and mobility in the network structure, other than the effect of vitrification on reaction kinetics.^79–82^

In consequence, if it is assumed that the stress relaxation process is governed by the kinetics of the reverse reaction, the retro Diels–Alder reaction, then the same set of rate constants that are valid for the modeling of the cross-linking process should be valid for the modeling of stress relaxation processes. Considering different reaction kinetics for the *endo* and *exo* adducts, we can write

$$\begin{array}{c} \frac{dx_{endo}}{dt}=-f_{\mathrm{eff}}\cdot {k^{\prime }}_{\mathrm{rDA},endo}\cdot x_{endo}\;\frac{dx_{exo}}{dt}=-f_{\mathrm{eff}}\cdot {k^{\prime }}_{\mathrm{rDA},exo}\cdot x_{exo}\\ \frac{dx}{dt}=\frac{dx_{endo}}{dt}+\frac{dx_{exo}}{dt}=-f_{\mathrm{eff}}\cdot ({k^{\prime }}_{\mathrm{rDA},endo}\cdot x_{endo}+{k^{\prime }}_{\mathrm{rDA},exo}\cdot x_{exo}) \end{array}$$ 22

For the sake of simplicity, it is assumed that the effectiveness factor *f*_eff_ in both reactions is the same. These rate equations are numerically integrated starting at *t* = 0 from the initial equilibrium values of *x*_*endo*_ = *x*_*endo*,eq_ and *x*_*exo*_ = *x*_*exo*,eq_ at a given temperature *T*. In each integration step, the network is analyzed depending on the global extent of conversion *x* = *x*_*endo*_ + *x*_*exo*_, in order to calculate the distribution of connected species and the structural parameters required for the analysis of the stress relaxation process, in a similar way to the general M–M method based on a single process. The detailed algorithm can be found in the Supporting Information.

#### Phenomenological Analysis

Making use of vertical shifts, the experimental and calculated stress relaxation curves can be fitted to stretched exponential functions,^15^ such as

$$\frac{\sigma }{\sigma_{0}}=\exp\left(-{\left(\frac{t}{\tau }\right)}^{\beta }\right)$$ 23

where σ is the stress, σ_0_ is the initial stress (providing the vertical shift factor used to normalize the data), τ is the characteristic relaxation time of the process, defined as the time needed to reach a fractional stress σ/σ_0_ = exp (−1) ≃ 0.368, and β is the stretching parameter, which defines the shape and the width of the relaxation time distribution. τ was determined by interpolation of the experimental data and model-calculated relaxation curves. Fitting was performed by minimizing the residual standard error (RSE) using β as single adjustable parameter. The RSE was calculated as

$$\mathrm{RSE}=\sqrt{\frac{\sum (y_{i}-y_{i,\mathrm{fit}}{)}^{2}}{n-1}}$$ 24

where *y*_*i*_ are the experimental or model-calculated normalized stress at a given time *t*_*i*_, *y*_*i*,fit_ are the values of the stretched exponential function calculated at *t*_*i*_, and *n* is the number of data points used in the fitting process. Data points with values of normalized stress between 0.99 and 0.01 and at regular intervals of 0.01 were interpolated and used in the analysis.

In this work, the stretched exponential model is mainly used with the purpose of determining the characteristic relaxation time τ and the width of the distribution of relaxation times, given by β, of experimental and model-calculated relaxation curves.

Relaxation times can be fitted by linear regression to an Arrhenius-like temperature dependence as

$$\ln\;\tau =C+\frac{E_{a}}{R\cdot T}$$ 25

where *E*_a_ is the apparent activation energy of the stress relaxation process.

Further analysis of the distribution of relaxation times has been carried out. An average relaxation time ⟨τ⟩ has been calculated as follows:^15,83^

$$\langle \tau \rangle =\frac{\tau \cdot \Gamma \left(\frac{1}{\beta }\right)}{\beta }$$ 26

The activation energy of the stress relaxation process has also been calculated using ⟨τ⟩ instead of τ in eq 25.

## 3 Experimental Section

### 3.1 Materials

The reversible polymer networks investigated in this study were synthesized by reacting a multifunctional furan-functionalized Jeffamine with a multifunctional maleimide, both containing oligomeric poly­(propylene oxide) spacers. The furan-functionalized Jeffamine 4F230 (molar mass 851 g/mol, *f* = 4.0)^33^ was prepared by functionalizing poly­(propylene glycol) bis­(2-aminopropyl ether) (Jeffamine D230, molar mass 282 g·mol^–1^, *f* = 4.0, Huntsman) with furfuryl glycidyl ether (molar mass 154 g·mol^–1^, purity 96%, *f* = 1.0, Sigma-Aldrich) using a previously established procedure.^30^ An amount of 1%w of the radical inhibitor 4-*tert*-butylcatechol (Sigma-Aldrich) was dissolved at 70 °C in the obtained compound to limit the occurrence of side reactions involving maleimide groups.

A fresh reactive mixture was prepared by mixing the resulting 4F230 compound with a stoichiometric amount of either bismaleimide 2M230 (molar mass 384 g·mol^–1^, functionality = 2.1) or trismaleimide 3M (molar mass 661 g·mol^–1^, functionality = 2.65),^33^ both purchased from Specific Polymers, at room temperature for 1 min. The starting reagents are depicted in Figure 3. The resulting polymer/network structures are illustrated in Schemes S1 and S2 (see the Supporting Information).

**Figure 3:**
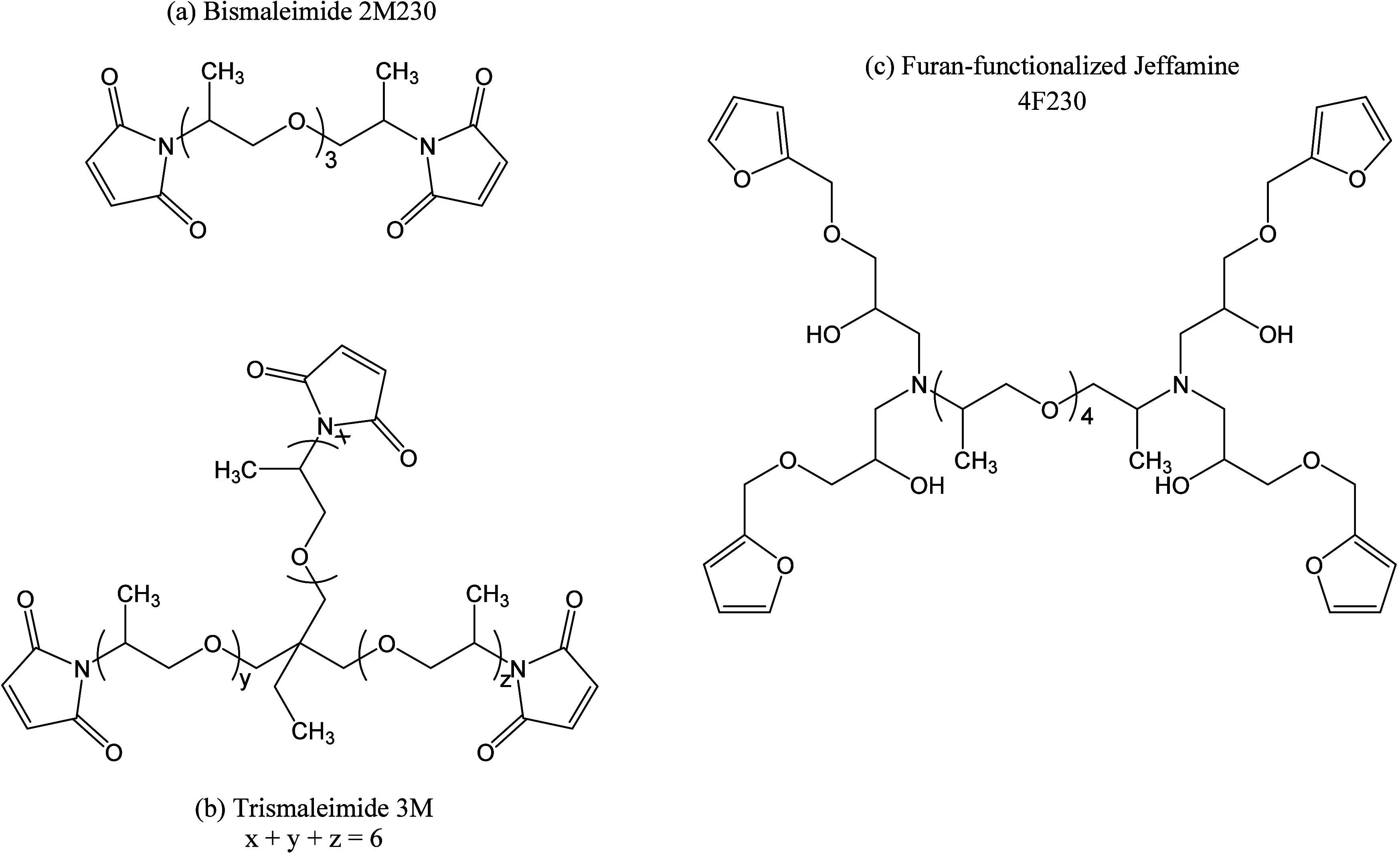
Structures of the furan and maleimide reagents used in this work.

### 3.2 Rheological Analysis

Stress relaxation curves were measured by dynamic rheometry. For the 4F230-2M230 system, a Discovery HR-2 hybrid rheometer (TA Instruments) equipped with an environmental test chamber (ETC) for precise temperature control was used with disposable aluminum parallel plates of 10 mm diameter. For the 4F230-3M system, an Anton Paar MCR 302 equipped with a CTD 450 chamber for temperature control was used with 10 mm parallel plates. Isothermal gelation experiments were performed between 30 and 80 °C, starting from a freshly mixed reactive mixture. The multifrequency mode was used at 0.3, 0.6, 1.0, 1.8, and 3.1 Hz with strains from 10 to 0.3%, automatically decreasing during the cure progress, as described in literature.^9^ For the stress relaxation experiments, freshly mixed network samples were positioned between the parallel plates at the initial measurement temperature (80 °C) and allowed to cure until equilibrium was reached, as determined by time sweep measurements. Stress relaxation was then monitored at a strain of 1% until full relaxation occurred. The samples were subsequently heated in 10 K increments, equilibrated, and the stress relaxation measurement repeated under the same conditions. Measurements were conducted across a temperature range of 80–120 °C for both networks. The effect of the applied initial strain on the stress relaxation kinetics was tested isothermally at 90 °C for strains between, 0.1 and 5%. All rheological measurements were performed within the linear viscoelastic regime, ensuring strain amplitude independence of the measured parameters, as confirmed by strain amplitude sweeps.

## 4 Results and Discussion

### 4.1 Analysis of the Kinetic-Structural Stress Relaxation Model

The features of the proposed analytical model and the validity of the general M–M model used for the analysis of stress relaxation processes will be discussed first in general terms.^70^ In Figure 4a the evolution of the concentrations of the different species were calculated using the analytical model for a generic, fully cross-linked, A_4_–B_2_ network. It can be clearly seen that the cross-links *A*_4,net_ are downgraded to lower functional species *A*_3,net_, eventually turning into linear units *A*_2,net_, which finally disappear from the system due to the deactivation of network strands along with *B*_2,net_ units. It is also observed that the number of units in the network strands *L* grows, which increases the impact of further bond exchange events, but the probability of bond exchange decreases due to the decreasing concentration of *B*_2,net_ fragments in the network structure.

**Figure 4:**
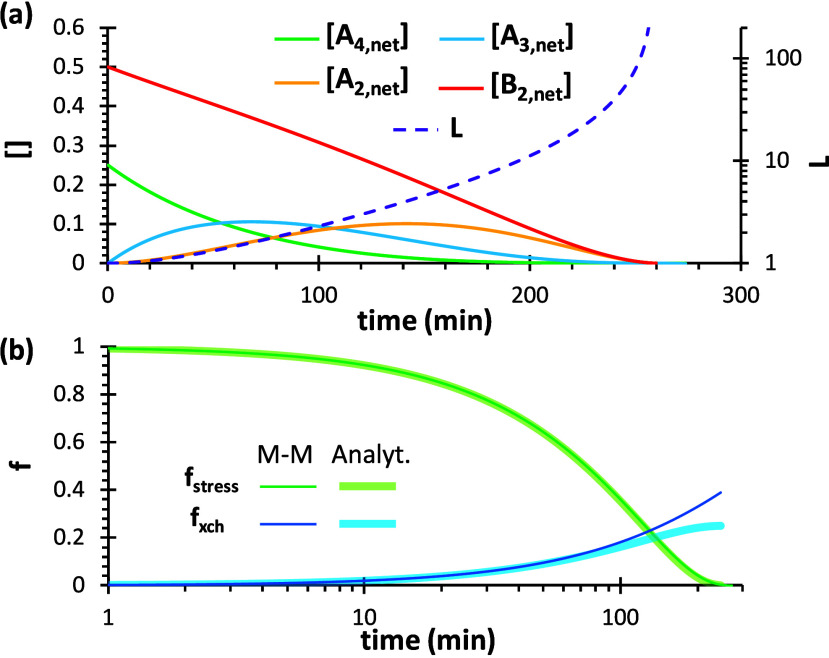
Outputs of the analytical model for A_4_–B_2_ networks, showing (a) evolution of the different species and average network strand length *L*, (b) calculated relaxation stress *f*_stress_ and fraction of bond exchange events *f*_xch_, in comparison with the M–M model with *f*_eff_ = 1. A total concentration of A and B groups equal to 1 (mol/kg) has been selected. A bond exchange rate constant of *k*_xch_ = 0.002 min^–1^ has been used for the calculations.

The calculated fractional stress, *f*_stress_ (eq 17), from the analytical model, shows a typical stress relaxation behavior in Figure 4b. In Figure 4b we also plot the stress relaxation curve that is predicted by the M–M model using *f*_eff_ = 1 (without correction for effectiveness) and the same bond exchange rate constant. It can clearly be observed that both models produce an equivalent result, which means that the M–M model is describing the same phenomena that we have discussed in the definition of the analytical model. In the figure we can also observe that he M–M method predicts that 42% of the bonds need to be exchanged for the A_4_–B_2_ network (gel point conversion is ca. 58%),^70^ while the analytical model predicts that only 25% of bonds need to be exchanged to relax the network. A likewise result is obtained for an A_3_–B_2_ network (not experimentally studied in this work), for which the M–M method predicts 29% of bonds need to be exchanged (gel point conversion is ca. 71%), while the analytical model predicts only 17% of the bonds need to be exchanged (see Supporting Information, Figure S1). Noteworthy, in an A_4_–B_2_ network there are 0.25 EANS per network bond, and in an A_3_–B_2_ network this ratio is also 0.17 (see model details in the Supporting Information). Indeed, the evolution of the cumulative effective bond exchange events and the decrease in EANS in the structure follow exactly the same trend (see Supporting Information, Figure S2). This implies that each effective bond exchange event deactivates one EANS in the network structure. This result aligns well with the general hypotheses made by other researchers,^53,55^ but they did not consider correctly the impact of bond exchange on the deactivation of network strands and elements within, and therefore the kinetics of the stress relaxation process were not correctly modeled.

Even though the M–M method calculates the total number of bond exchange events, some of these take place within deactivated strands, not impacting the stress relaxation. Given that the result is equivalent to the analytical model that considers only the number of effective bond exchange events, it can be concluded that the M–M method provides a mechanistic modeling solution for this de-cross-linking-like process, as it correctly describes the effect of deactivating network strands in the course of a stress relaxation process. This method is preferred over the analytical model for its flexibility in terms of the wide range of network architectures that can be analyzed and its inclusion of the effect of permanent bonds and complex relaxation kinetics.^70^

The common assumption that the stress relaxation is equivalent to a Maxwell relaxation (equivalent to stretched exponential with β = 1) needs to be discussed. Previous theoretical approaches are based on the consideration that the rate of bond exchange is equivalent to the rate of disappearance of network strands or cross-links,^53–55^ leading to Maxwell stress relaxation kinetics. However, such equivalence cannot be justified in terms of the impact of a bond exchange event on the network structure,^70^ as recently discussed. Moreover, it needs to be taken into consideration that many relaxation processes show values of β consistently lower than one.^15,17,18,56^ The stress relaxation calculated using the analytical and the M-M models was fitted to a stretched exponential model, yielding a value of β ≃ 1.2 > 1 (see Figure S3 in Supporting Information, entry *b*/*RT* = 0, for details). Assuming that our modeling approach is more correct from a structural perspective, the elevated value of β ≃ 1.2 evidence the need for an effectiveness factor *f*_eff_ to obtain a more correct representation of the stress relaxation curve.^70^

In the previous work by Konuray et al.,^70^ it was stated that bond exchange events become less effective due to the increase in chain segments in the material that had not yet been exchanged but had nevertheless been deactivated as seen in Figure 1, prompting an approximate definition of the efficiency factor analogous to eq 20. However, Figure 4b shows that the stress relaxation kinetics calculated with the M–M model using *f*_eff_ = 1 (without correction for effectiveness) already takes into consideration that some of bond exchange events are not going to have an impact in terms of stress relaxation. Therefore, it is evident the justification for *f*_eff_ from that earlier work^70^ is not valid. The need for such a correction factor to produce more realistic stress relaxation curves should be argued in different terms.

An improved effectiveness factor *f*_eff_ was formally defined in Section 2.3, eq 21, which considers the decreased activation energy for bond exchange in the early stages of the stress relaxation process. To evaluate this effect, Figure 5 shows stress relaxation curves simulated for a fully cross-linked A_4_–B_2_ system with varying values of the dimensionless parameter *b*/*R*·*T*. A number of interesting trends are revealed. First, Figure 5a shows that increasing the value of *b*/*R*·*T*, thus reducing the apparent activation energy, produces a noticeable decrease of the relaxation time, along with a significant increase in the width of the stress relaxation process. When *b*/*R*·*T* = 0 (*f*_eff_ = 1, no correction to the relaxation curve), the relaxation process hardly spans two decades, while in the case of *b*/*R*·*T* = 8, it extends three to four decades. Second, this simple bond exchange process ends when “degelation” is reached (Figure 5b), i.e., when *x* ≃ 0.58, the gel point conversion for an A_4_–B_2_ system. Third, it can be observed that increasing values of *b*/*R*·*T* lead to higher initial values of *f*_eff_, which eventually decrease to one at a faster rate in the course of the stress relaxation process, as shown in Figure 5c, because of the decreasing fraction of dynamic bonds contributing to stress in the process. Figure 5a also shows a comparison with an older definition of *f*_eff_ reported in the previous work.^70^ It can be observed that, although the shape is similar to those obtained when *b*/*R*·*T* is in the range 2–4, the effect is opposite: a delay in the stress relaxation process. Moreover, the shape cannot be changed. The comparison of the improved *f*_eff_ defined in this work over the old definition will be expanded in later sections.

**Figure 5:**
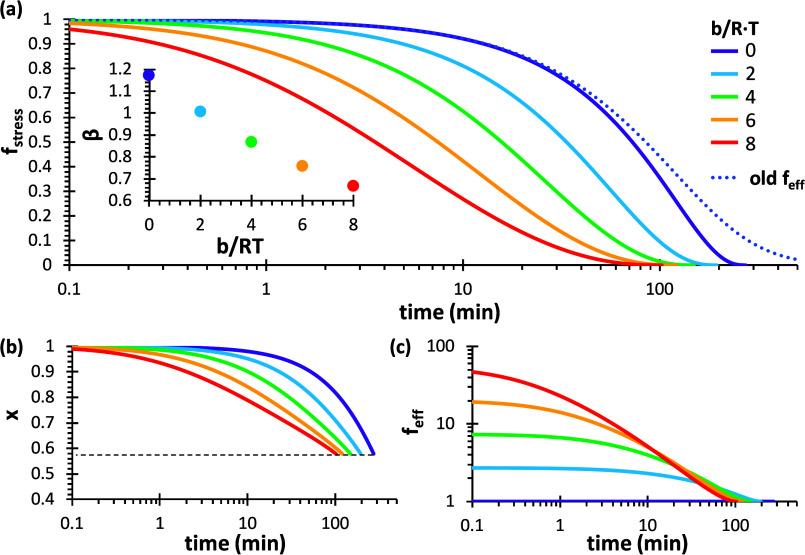
Analysis of the effect of the dimensionless parameter *b*/*R*·*T* on the stress relaxation kinetics of an A_4_–B_2_ system using the M–M model: (a) stress relaxation curves (the inset shows the parameter β corresponding to the fitting to a stretched exponential function); (b) extent of conversion *x*; and (c) effectiveness factor *f*_eff_. A bond exchange constant *k*_xch_ equal to 0.002 min^–1^ was used for the calculations. A comparison with an older definition of *f*_eff_, dotted line, is also shown in (a).

To illustrate that the novel M–M model provides a relevant relaxation behavior, a single Maxwell model with stretched exponential was fitted onto the calculated curves of Figure 5a (see Figure S3 in Supporting Information for details). Stretched exponential models^15^ or multiple Maxwell elements are currently the best practice in literature for the fitting of broad distributions of single relaxation processes or multiple relaxation processes, respectively.^14,58^ In this work, we only use the stretched exponential model with the purpose of determining the characteristic relaxation time τ and the width of the distribution of relaxation times, given by β. Stress relaxation experiments of CAN reported mainly values of β between 0.5 and 1.^15,17,56–58,84,85^ most commonly between 0.75 and 1 for simple relaxing systems. Extremely low values of β of 0.15 have also been reported for certain systems.^86^ For the stretched exponentials fitted to the M–M model curves, the shape parameter β decreases from values of ca. 1.2 for *b*/*R*·*T* = 0 (undisturbed bonds) to ca. 0.65 for *b*/*R*·*T* = 8 (a strongly reduced bond strength for stressed bonds), all in fair agreement with the reported range of β values. Broad stress relaxation processes with lower values of β have often been interpreted as an indication of a complex distribution of relaxation times or the coexistence of bonds with different exchange kinetics. However, our modeling results suggest there is an apparent distribution of relaxation times due to the impact of the stress-induced lowering of the activation energy for the breaking of the dynamic bonds within the network structure. The kinetics and shape of the relaxation process are clearly related to the definition of *f*_eff_: the higher the value of *b*/*R*·*T*, the higher is the initial value of *f*_eff_ and the faster is the initial bond exchange. However, for the same reason, the impact of the decrease in elastic activity as the relaxation advances is more pronounced, slowing down the relaxation process. The combined effect of faster relaxation at the beginning and the slowing down at the end result in a relaxation profile with a low β value. Following the same reasoning, lower values of β should be obtained with more densely cross-linked systems, with a higher number of dynamic bonds in the EANS, *n*_(+),EANS_, and thus a higher initial *f*_eff_.

The question arises whether or not the value of the energetic disturbance *b* has any physical meaning and whether or not it attains reasonable values. If one assumes that simple relaxation processes would have typical values of β around 0.75–1, this implies that *b*/*R*·*T* should be around 2–6 for a simple A_4_–B_2_ system (see Figure 5a). Assuming a relaxation temperature around 400 K, the value of *b* would be around 6–20 kJ/mol. These magnitudes do not seem unreasonable given that bond exchange activation energies can range between ca. 50 and 250 kJ/mol^14,15,86–88,17,18,25,26,54,56,84,85^ although lower values have also been reported.^26^ Given that the decrease in activation energy can produce such a strong impact on *f*_eff_ (see Figure 5c) and therefore on the kinetics, the justification of *b* is imperative.

In order to determine whether the proposed model and the definition of the effectiveness factor *f*_eff_ are valid, the model outputs must be compared with experimental stress relaxation data for which the underlying bond exchange kinetics is independently available. This is critical because a vast majority of kinetic data from stress relaxation processes are derived directly from experiments. We choose to analyze furan-maleimide Diels–Alder dynamic networks for which complete cross-linking kinetics data are available, obtained after a careful numerical fitting of a wide range of compositions and experimental conditions.^33^ Assuming that the bond exchange kinetics is determined by the reverse reaction,^25,70^ the values of the retro Diels–Alder constants reported in the literature^33^ could be used directly as bond exchange constants. This permits testing whether or not the definition of the kinetic model is reasonable and whether or not it has qualitative and/or quantitative predictive capabilities. Diels–Alder systems are further complicated due to the temperature-dependent equilibrium, which makes it necessary to consider the starting equilibrium network at the beginning of the relaxation process. In consequence, the accuracy of the kinetic-structural model for the prediction of the cross-linking kinetics and the network build-up process will have to be validated as well.

### 4.2 Comparison of Model Outputs with Experimental Results

#### Cross-Linking Kinetics

In a previous work, the reaction kinetics of different furan-maleimide Diels–Alder networks were studied in detail, and a consistent set of curing kinetic parameters valid for a wide range of networks was determined from DSC analysis.^33^ Gel times were determined from isothermal curing experiments in multifrequency dynamic rheometry and compared with isothermal reaction kinetics in order to determine the gel point conversion *x*_gel_ at different curing temperatures. Figure 6a shows an example of the gel time determination for a 4F230-2M230 network and Figure 6b summarizes the calculated gel point conversions for 4F230-2M230 and 4F230-3M networks, in addition to the average values for each network.

**Figure 6:**
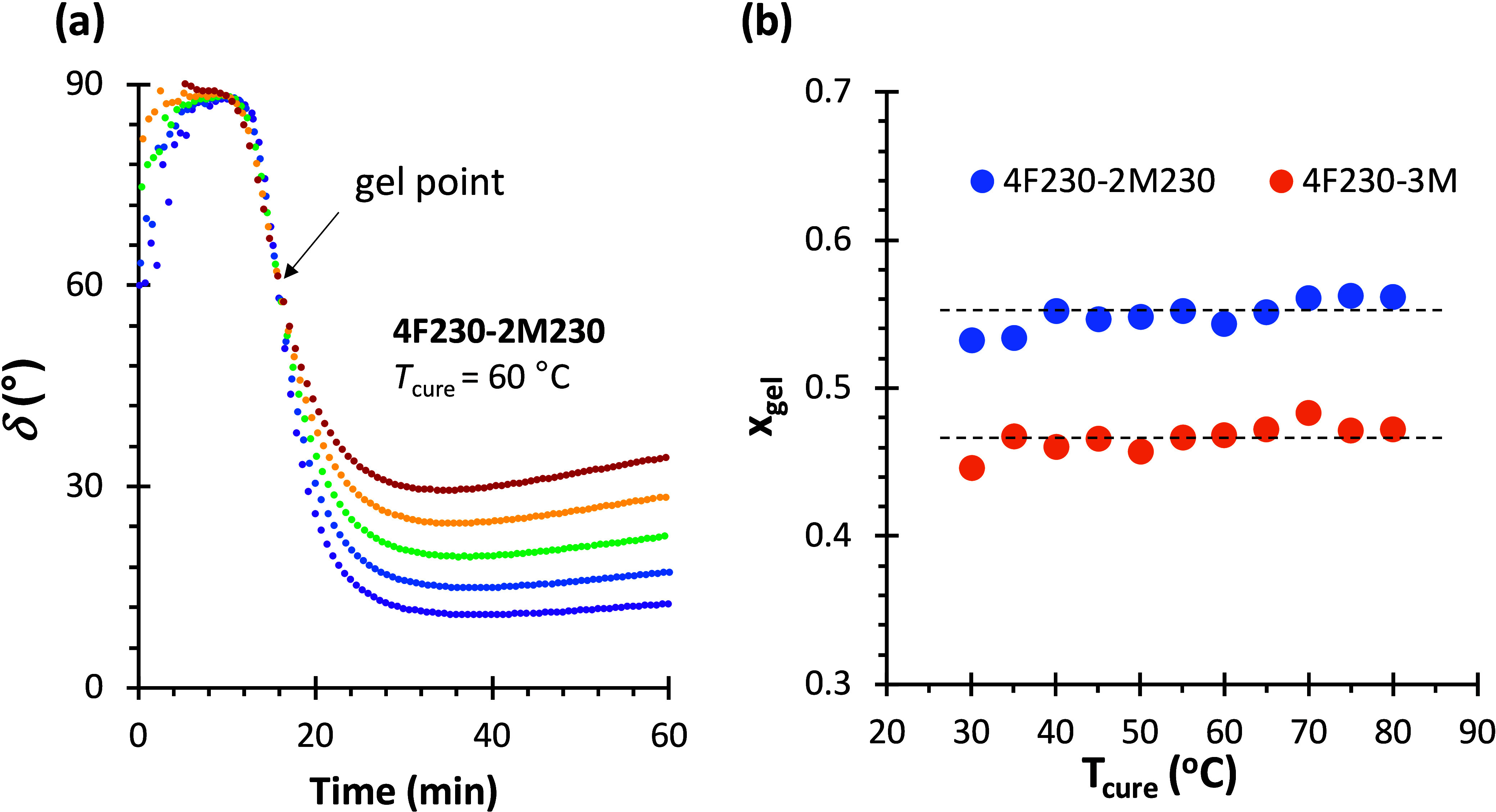
(a) Gel time determination from a multifrequency isothermal curing experiment in dynamic rheometry, frequency range from 0.31 to 3.12 Hz; (b) gel point conversion calculated for 4F230-2M230 (average 0.55, standard deviation 0.01) and 4F230-3M formulations (average 0.47, standard deviation 0.01).^33^

Assuming that 4F230-2M230 and 4F230-3M networks are equivalent to A_4_–B_2_ and A_4_–B_3_ systems respectively, the average values are in fair agreement with the theoretical gel point conversion of 58% and 41% predicted for an ideal step-growth network polymerization using the well-known Flory–Stockmayer expression (see Section 2.2 and eq 8). Taking into consideration experimental uncertainty, and for the sake of simplicity, the purity of the starting components and other deviations from the ideal step-growth behavior such as the occurrence of intramolecular loop formation^89,90^ will be ignored. The validity of these assumptions will be discussed later.

The conversion curves of 4F230-2M230 at different temperatures have calculated using the kinetic model and represented in Figure 7a, showing clearly that the equilibrium conversion attained at the end of the reaction decreases with increasing temperature. The theoretical gel times have been determined from the calculated curves and the theoretical conversion at gelation of 58%. The evolution of the cross-linking density, represented by the EANS, has been calculated using the M-M model and represented in Figure 7b. It is evidenced that, while the equilibrium conversion varies mildly with temperature, decreasing from 0.90 at 80 °C to 0.74 at 120 °C), the equilibrium cross-link density strongly depends on temperature, showing a 4-fold decrease.

**Figure 7:**
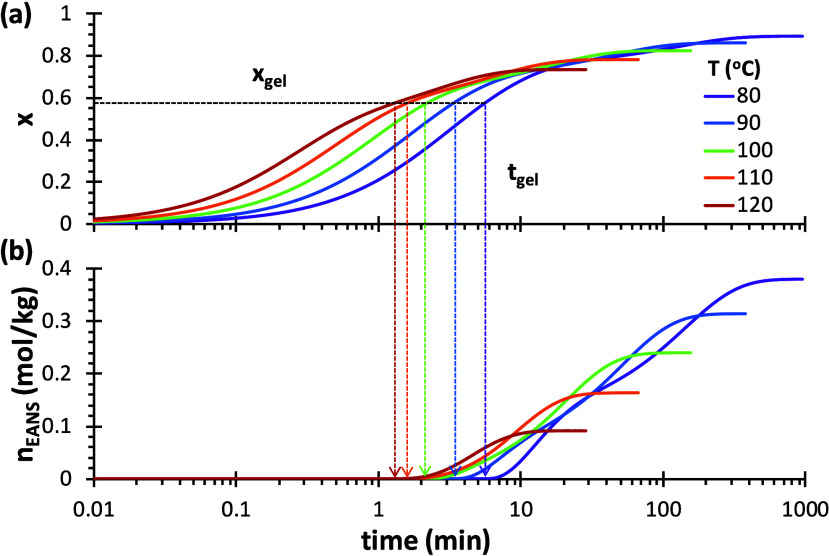
(a) Simulation of (a) *x*–*t* curves for the curing process of 4F230-2M230 at different temperatures, indicating the theoretical gelation time, and (b) evolution of the cross-linking density, given by the number of EANS, for the same system, showing the temperature-dependent equilibrium structure reached.

A remarkable agreement is observed when comparing the experimental evolution of G’ during the cross-linking of 4F230-2M230 with the simulated evolution of EANS calculated using the kinetic-structural model (Figure 8a). Gelation is observed around 3.5 min. The cross-linking curves show clear changes in slope around 10 min due to the shift from the kinetically favored *endo* adduct to the thermodynamically stable *exo* adduct, and the attainment of an equilibrium network structure. In Figure 8b, a roughly proportional relation was found between the equilibrium moduli *G* at different temperatures and the calculated equilibrium *n*_EANS_. Similar results were obtained for 4F230-3M in Figure 9. The proportionality between *G* and *n*_EANS_ is more evident when the temperature (eq 9) is taken into consideration (see Figure S4 in Supporting Information).

**Figure 8:**
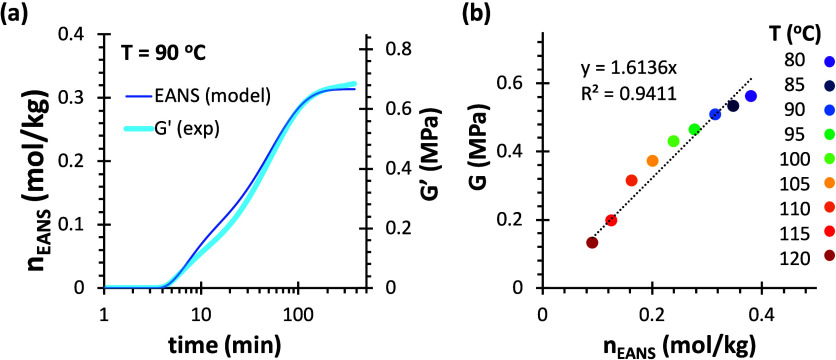
(a) Comparison of the evolution of *G*′ during cross-linking of 4F230-2M230 at 90 °C with EANS calculated using the kinetic-structural model. (b) Comparison of equilibrium moduli *G* of 4F230-2M230 (determined from the equilibrium relaxation moduli at the beginning of stress relaxation experiments) and *n*_EANS_ calculated using the model; the dashed line shows the linear regression with intercept at the origin.

**Figure 9:**
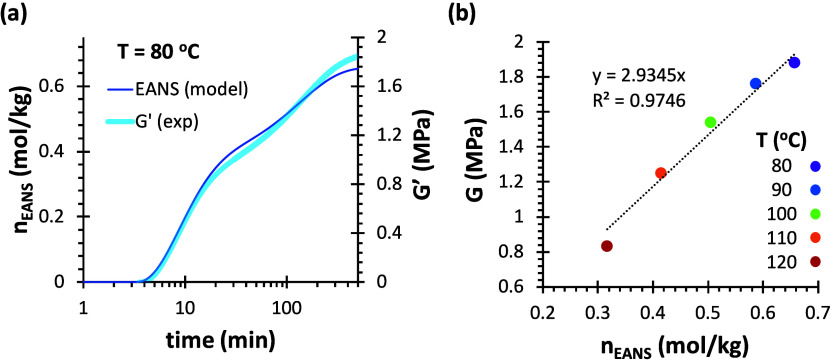
(a) Comparison of the evolution of *G*′ during cross-linking of 4F230-3M at 80 °C with EANS calculated using the kinetic-structural model. (b) Comparison of equilibrium moduli *G* of 4F230-3M (determined from the equilibrium relaxation moduli at the beginning of stress relaxation experiments) and *n*_EANS_ calculated using the model; the dashed line shows the linear regression with intercept at the origin.

These results evidence the general validity of the proposed cross-linking model, based on the ideal step-growth polymerization behavior and the phantom network model, by which *G* ∝ *n*_EANS_ (eq 9). The discrepancy between the proportionality constant of both systems can be explained by the experimental conditions used: a gap of 170 μm was used for the rheological analysis of 4F230-2M230 network, while a gap of 500 μm was used for the 4F230-3M network. Given that low values of gap would introduce a higher uncertainty in the value of the measured moduli, additional rheological experiments were carried with 4F230-2M230 network out, with a gap of ca. 500 μm, to cross-check the validity of the data and the modeling. The results (see Figure S5 in the Supporting Information) confirm the validity of the cross-linking kinetics modeling (Figure S5a), and the proportionality between *G* and *n*_EANS_, but yielding higher values of moduli and therefore a value of the proportionality constant now more similar to the 4F230-3M network (compare Figures 9b and S5b). It can therefore be concluded that the network build-up model describes correctly the cross-linking process and the temperature-dependent equilibrium network structure, and that there are no relevant differences between the two polymer networks other than the cross-linking density, depending on the functionality of the maleimide component.

However, Figures 8a and 9a also reveal the measured modulus *G*′ and *n*_EANS_ are not strictly proportional, with *n*_EANS_ rising somewhat faster than *G*′ at the beginning of the cross-linking and *G*′ growing faster than *n*_EANS_ toward the end of the cross-linking process. At these low temperatures, the occurrence of side reactions leading to further cross-linking and deviations from the proposed network build-up model is not very likely, so there must be some deviations probably caused by either the assumption of ideal step-growth cross-linking behavior and the change in the proportionality constant between *G* and *n*_EANS_ due to the changes in the topology and mobility of the network structure taking place in the cross-linking process, or else to changes in the network stiffness depending on the contribution of *exo* or *endo* adduct structures. All these effects might also be the cause for the limited nonlinearity between *G* and *n*_EANS_ also observed in Figures 8b and 9b. It is therefore acknowledged that this might introduce some uncertainty in the predictive capabilities of the stress relaxation model.

#### Stress Relaxation Kinetics

Experimentally, it was first verified for the 4F230-2M230 network that the relaxation times were not affected by the magnitude of the applied strain up to the upper limit of 5% (Figure 10a), which was not unexpected.^7^ Stress relaxation experiments at different temperatures show that the initial modulus decreases with increasing *T* (Figure 10b). As discussed before, this effect was correctly described by the decreasing *x*_eq_ with increasing temperature, leading to lower values of model-calculated *n*_EANS_ (see Figure 8b). Overall, it can be observed that the different experiments cover a broad temporal range, spanning about 4 decades. Experimental relaxation curves of 4F230-3M network at different temperatures show similar trends (see Figure S6a in Supporting Information).

**Figure 10:**
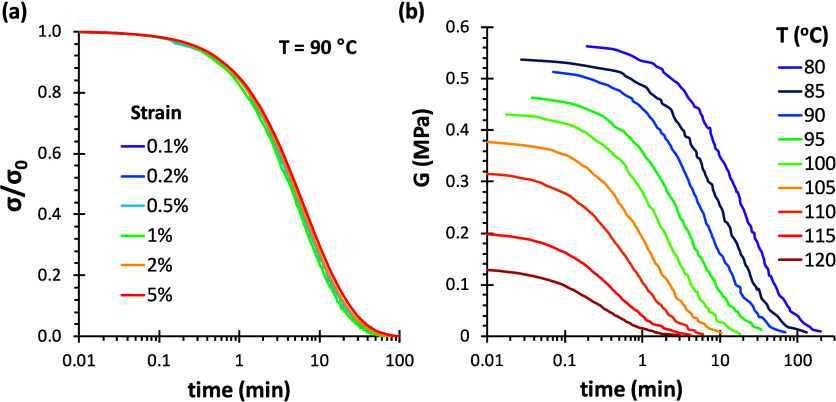
Experimental stress relaxation curves of 4F230-2M230 networks (a) at 90 °C and different strain and (b) with a fixed strain of 1% and at different temperatures.

As a point of reference for the kinetic-structural relaxation model, the stress relaxation curves were normalized with respect to the initial modulus^25,26,28^ and fitted the curves to a stretched exponential model in order to determine characteristic relaxation time and width of the relaxation. The results are shown in Figure 11 for the 4F230-2M230 network and Figure S6b for the 4F230-3M network (see Supporting Information). It was verified the excellent reproducibility of the kinetics of stress relaxation data, regardless of the gap size (see Figure S7 in the Supporting Information). The fitting parameters are shown in Table 1 for 4F230-2M230 network (detailed fitting data in Table S2 in Supporting Information) and Table 2 for 4F230-3M network (detailed fitting data in Table S3 in Supporting Information). The agreement between the experimental and fitted curves in Figure 11 is excellent. The inset in Figure 11 shows the derivation of the apparent activation energy of the relaxation process for the 4F230-2M230 network by linear regression. The values in Tables 1 and 2 show that the activation energies of the stress relaxation processes for the two networks are very similar to the activation energy of the retro Diels–Alder reactions (113.1 and 123.6 kJ·mol^–1^ for the *endo* and *exo* adducts, respectively),^33^ which was also expected.^25,70^ Interestingly, the stretching parameter β attained values in the range 0.8–0.9, with a clear increasing trend with increasing *T* for both 4F230-2M230 and 4F230-3M networks. If one compares the results in Table 1 and Table 2, it is also evident that the stress relaxation process is considerable slower for the 4F230-3M network, having relaxation times twice as high as those of the 4F230-2M230. The average relaxation times ⟨τ⟩ have been calculated (see Tables S2 and S3 in Supporting Information), leading to values only moderately higher than τ because the distributions of relaxation times are quite narrow. The activation energy of the stress relaxation process determined from the values of ⟨τ⟩ are also very similar to those obtained from τ.

**Figure 11:**
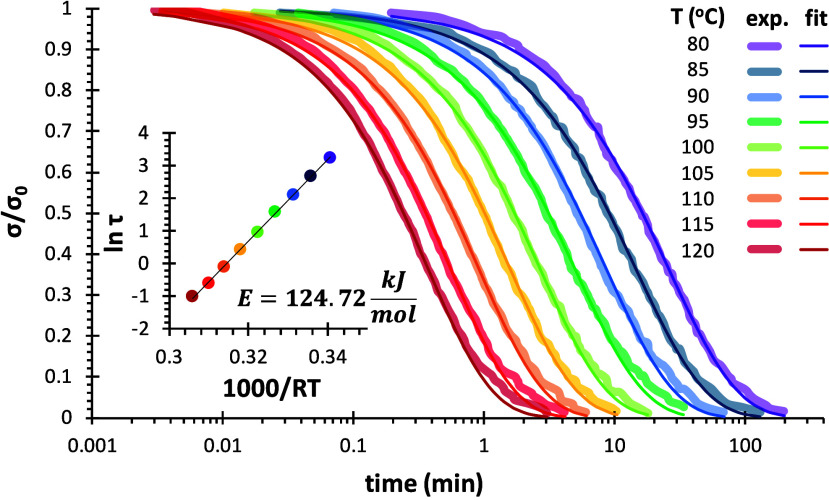
Fitting of the normalized experimental stress relaxation curves of 4F230-2M230 system to the stretched exponential model. The inset shows the determination of the activation energy from the relaxation times τ.

**Table 1:** Summary of the Experimental Relaxation Parameters of 4F230-2M230 Network and the Results of the Modeling Approaches^a^

			model					
	experimental		M–M		*b* = 11.5		*b* = 12 + att.	
*T* (°C)	τ (min)	β	τ (min)	β	τ (min)	β	τ (min)	β
80	25.5	0.82	66.4	0.97	25.6	0.80	25.5	0.77
90	8.5	0.82	18.3	1.00	7.98	0.84	8.05	0.82
100	2.66	0.83	5.19	1.02	2.59	0.89	2.68	0.86
110	0.90	0.85	1.49	1.06	0.87	0.94	0.93	0.90
120	0.37	0.87	0.42	1.08	0.29	1.00	0.34	0.93
*E*(kJ/mol)	124.7		145.8		126.8		123.9	

a Additional data and plots are given in Table S2 and Figure S8 in the Supporting Information.

**Table 2:** Summary of the Experimental Relaxation Parameters of 4F230-3M Network and the Results of the Modeling Approaches^a^

			model					
	experimental		M–M		*b* = 11.5		*b* = 12 + att.	
*T* (°C)	τ (min)	β	τ (min)	β	τ (min)	β	τ (min)	β
80	54.2	0.77	147.2	0.95	53.6	0.79	54.7	0.75
90	16.60	0.79	42.2	0.96	16.7	0.80	17.3	0.77
100	5.20	0.80	12.6	0.97	5.50	0.82	5.74	0.78
110	1.73	0.83	3.87	0.99	1.87	0.85	2.00	0.80
120	0.61	0.82	1.21	1.00	0.66	0.89	0.73	0.83
*E*(kJ/mol)	129.8		138.6		127.0		124.6	

a Additional data and plots are given in Table S3 and Figure S9 in the Supporting Information.

First, the predictive capabilities of the kinetic-structural approach were verified with the simplest relaxation model based on the M–M network analysis, assuming that there is no effectiveness factor (*f*_eff_ = 1). The model results for the 4F230-2M230 network are given in Figure 12. The initial point of the relaxation process is the temperature-dependent equilibrium conversion. The stress relaxation process always finishes at *x* = *x*_gel_, as described by the model. Consequently, the extent of bond exchange required for full relaxation is different depending on the temperature. It can be observed that the evolution of the calculated EANS in Figure 12b looks very similar to the experimental relaxation curves in Figure 10. However, the normalized stress relaxation curves of the model show significant differences with the experimental data (Figure 12c). The model curves were also fitted to the stretched exponential, with results shown in Table 1 (M–M column). It can be observed that the relaxation times are systematically overestimated, especially at lower temperatures. However, Figure 12c shows that the global time scale of the relaxation process is correctly estimated for all temperatures, which is evidence of the validity of the proposed kinetic-structural model. The increasing trend in β is correctly predicted, even though its values are overestimated. The activation energy is significantly overestimated, which is believed to be caused by the fact that the relaxation processes start at different equilibrium conversions depending on the temperature, but all end at the same “degelation” conversion.

**Figure 12:**
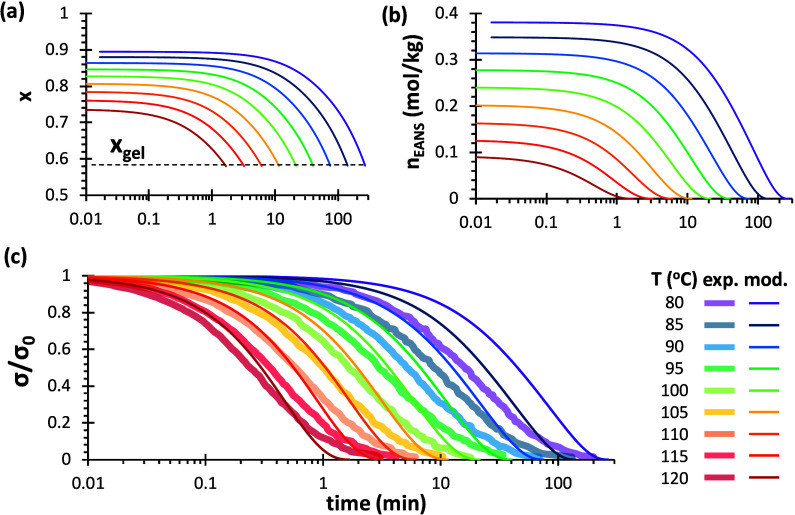
Simulation of the relaxation process of 4F230-2M230 network with the M–M model without considering the efficiency factor, that is, *f*_eff_ = 1. (a) Evolution of conversion *x* and (b) *n*_EANS_; (c) normalized stress relaxation in comparison with experimental data.

With regards to the 4F230-3M network, similar results were obtained, as seen in Figure S10 in Supporting Information). It should be highlighted that the application of the M–M model to the 4F230-3M network (Table 2, M–M column) produced relaxation times that are around twice those corresponding to the 4F230-2M230 network (Table 1, M–M column), following the same trend as the experimental data (compare Tables 1 and 2, Experimental column). This is explained by the fact that “degelation” is achieved at a conversion of 41% for the 4F230-3M network (see Figure S10a in Supporting Information), while this is accomplished at a conversion of 58% for the 4F230-2M230 network. It is therefore quite clear that it will require a higher extent of bond exchange and therefore a longer time for the 4F230-3M network to relax. Such effect was already predicted in previous work by Konuray et al.^70^ Even if there are substantial differences between the predicted curves and the experimental data, the global time scale of the relaxation process is also correctly estimated (see Figure S10b in Supporting Information).

The analysis of the results obtained from the application of the M–M model with *f*_eff_ = 1 to 4F230-2M230 and 4F230-3M networks indicates that structure-dependent kinetic trends can be predicted and global relaxation time scales can be correctly estimated. This constitutes a first evidence of the validity of the key model hypotheses, namely: (i) the degelation analogy for the description of the stress relaxation process, enabling the use of the M–M network model, and (ii) the control of the stress relaxation process by the reverse DA reaction.

To alleviate the limited prediction capabilities of this basic M–M model with *f*_eff_ = 1, the form of *f*_eff_ was revisited. The new definition of *f*_eff_, given by eq 21, accounts for an enhancement of exchange rate due to a decrease of the activation energy as a function of the stress exerted on the elastically active chains (factored in by a constant *b*).

Figure 13 shows the outputs of the application of the model to the relaxation process of 4F230-2M230 networks, making use of *f*_eff_ described by eq 21. A single value of *b* = 11.5 kJ/mol was used for all the temperatures. It can be clearly observed (Figure 13a) that the fraction of activated bonds *f*_(+),EANS_ is more elevated at lower temperatures, due to the decreasing conversion *x*_eq_ and cross-linking density, and so is the resulting *f*_eff_ (Figure 13b). In the course of the relaxation process, *f*_(+),EANS_ tends to 0 and *f*_eff_ tends to a value of 1 for all temperatures. The impact of *f*_eff_ is more relevant at lower temperatures and at the beginning of the relaxation process (see Figure 13c). A similar effect was also observed for de 4F230-3M network, using the same value of *b* = 11.5 kJ/mol (see Figure S11 in Supporting Information). Figure 14 shows that this improved effectiveness factor *f*_eff_ presents a drastic improvement of the predictive capabilities of the model, using a single value of *b* = 11.5 kJ/mol for both systems at all the tested temperatures. A remarkable agreement between the calculated and experimental stress relaxation curves is apparent. The onset of the stress relaxation process is correctly predicted in all cases. Observing the τ and β obtained by analyzing the model outputs (Tables 1 and 2, *b* = 11.5 column), a better match with the τ and β obtained by fitting the experimental results can be appreciated. The same can be stated for the predicted activation energy.

**Figure 13:**
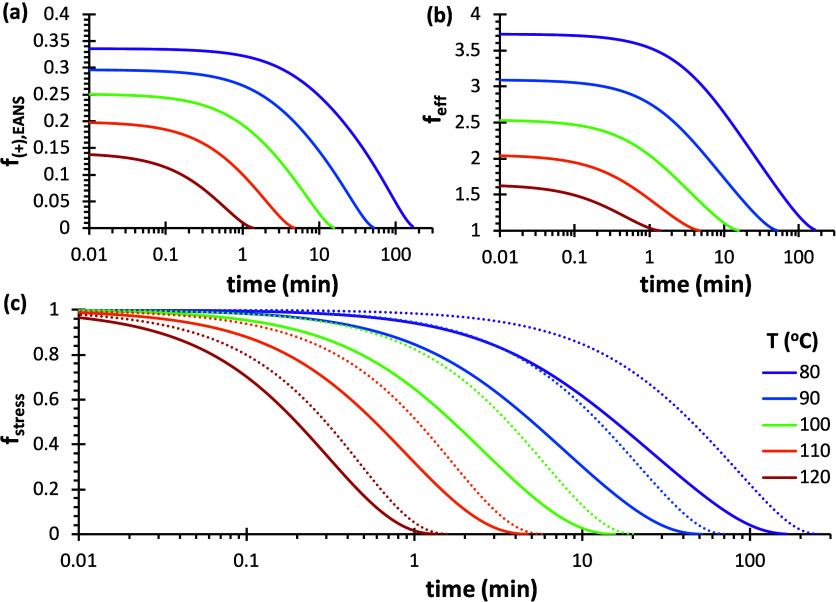
Detailed outputs of the modeling of the stress relaxation kinetics of 4F230-2M230 network using the M–M model with efficiency factor depending on *b* = 11.5 kJ·mol^–1^, showing detailed evolution of (a) *f*_(+),EANS_, (b) *f*_eff_ , and (c) *f*_stress_, in comparison with the M–M model with *f*_eff_ = 1 (dotted line).

**Figure 14:**
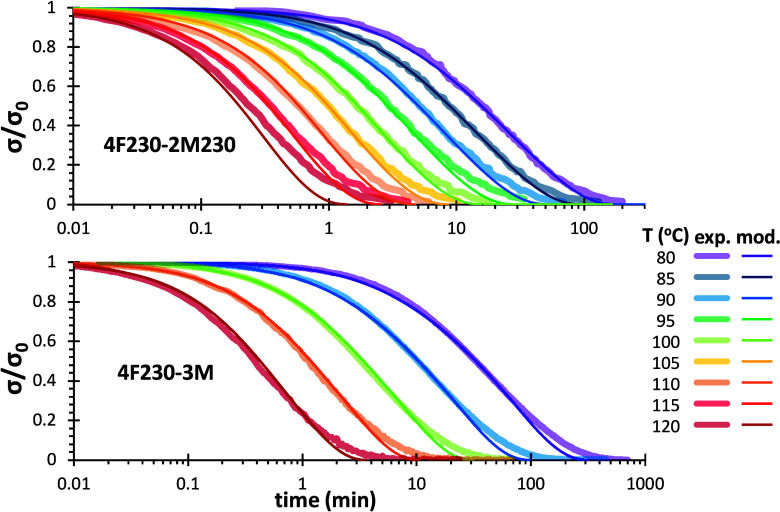
Simulation of the relaxation process of 4F230-2M230 network and 4F230-3M network with the M–M model with efficiency factor *f*_eff_ calculated with *b* = 11.5 kJ/mol. Normalized calculated stress relaxation curves are compared with experimental data.

Even though the relaxation curve shapes are accurately predicted, some discrepancies are observed at the end of the stress relaxation process, where the predicted relaxation rate is higher than the experimental one, and this especially for higher temperatures in the case of the 4F230-2M230 network. To remedy this, a reduced effectiveness of bond exchange events toward the end of the relaxation process was proposed, caused by the low fraction of dynamic bonds contributing to the remaining elastic response of the material at later stages of relaxation. An attenuation factor is incorporated into *f*_eff_ as shown in eq 27. The attenuation factor comprises a fitting parameter *a*. The multiplicative attenuation function would reduce *f*_eff_ toward the end of the relaxation, as the fraction of dynamic bonds contributing to the elastic activity of the network *f*_(+),EANS_ decreases:

$$f_{\mathrm{eff}}=\left[\exp\left(f_{(+),\mathrm{EANS}}\cdot \frac{b}{R\cdot T}\right)\right]\cdot \left[\frac{{f_{(+),\mathrm{EANS}}}^{2}}{a+{f_{(+),\mathrm{EANS}}}^{2}}\right]$$ 27

The results of this adjustment are shown in Figure 15 with *a* = 0.002 for the 4F230-2M230 network, and with *a* = 0.004 for the 4F230-3M network; the value of *b* was slightly increased to 12 kJ/mol for both networks, because of the presence of the attenuation factor. The shape of the stress relaxation curves is correctly reproduced, producing an excellent agreement between experimental and calculated data, which is especially evident for 4F230-2M230 network (compare Figure 14 with Figure 15). Noticeably, the impact of this correction is higher in the stress relaxation curves at higher temperatures, because of the lower starting cross-linking density and fraction of stressed dynamic bonds (see detailed outputs of the model in Figures S12 and S13 in Supporting Information). The fitting of the calculated curves to a stretched exponential model (see Tables 1 and 2, *b* = 12 + att. column) and the recalculated activation energies confirm these observations. The variation in the optimal value of parameter *a* can be explained by the difference in the structures of 4F230-2M230 and 4F230-3M networks. However, good the final result is, the physical significance of such attenuation factor is not clear yet. It may be hypothesized that, at long times and very low concentration of EANS contributing to the stress response of the material, the dissociation of the adduct itself is no longer the rate-limiting step and so the de-cross-linking analogy becomes less exact. The possibility that a dissociated species finds a different partner to produce an effective bond exchange, instead of reassociating with the original one, could be lower, therefore resulting in less effective release of the stress; mobility/diffusion limitations depending on the network structure could play a relevant part.^91^ The simple definition of the effectiveness factor, depending on activation parameter *b* and structural parameter *f*_(+),EANS_ could be revised in the light of these considerations. It would also be convenient to analyze more in-depth mechanochemical effects^61^ on the activation of dynamic bonds within the polymer chains. This field is widely open for research.

**Figure 15:**
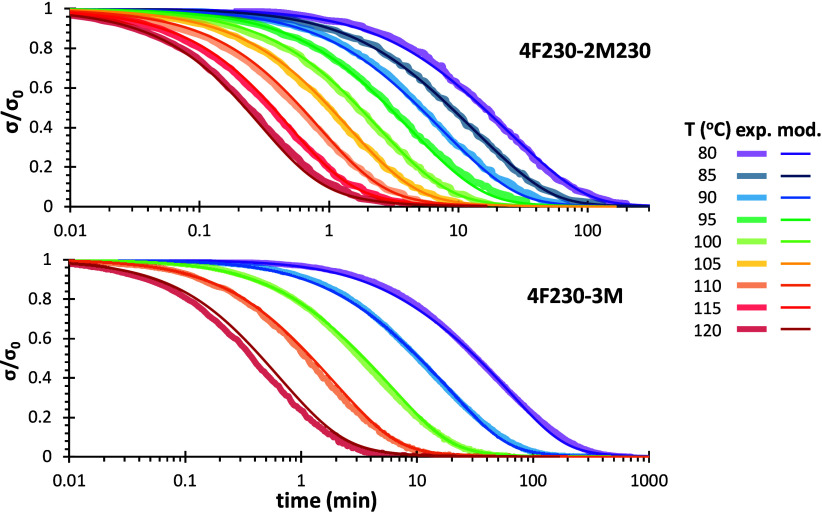
Simulation of relaxation processes of 4F230-2M230 and 4F230-3M networks using the final model and comparison with experimental data. An activation factor of *b* equal to 12 kJ·mol^–1^ was used for both networks. Attenuation is calculated with *a* = 0.002 for 4F230-2M230 network and =0.004 for 4F230-3M network.

## 5 Conclusions

The stress relaxation of CANs based on the reversible Diels–Alder (DA) chemistry was successfully modeled using a kinetic-structural model. This model is based on the analogy between stress relaxation and network de-cross-linking (degelation) where stress is relaxed due to (reversible) chain scission and the remaining stress is due to the remaining elastically active network strands (EANS). A recursive network analysis methodology, inspired by the well-known Macosko–Miller (M–M) method, was coupled with the kinetic model taking into consideration (i) the reaction kinetics of the retro Diels–Alder reactions, (ii) the increased activation of stressed dynamic bonds within the EANS, and (iii) the decreasing effectiveness of bond scission events toward the end of the relaxation process.

The prediction capabilities of the model were tested using a bottom-up approach. The kinetic-structural model was validated by means of the rheological characterization of the cross-linking and stress relaxation of two different Diels–Alder-based networks, for which complete reaction kinetics data were already available. The cross-linking process was correctly described by the combination of the M–M network build-up model and the kinetic model. The temperature-dependent modulus, which is related to the equilibrium network structure at the beginning of the stress relaxation process, is also correctly obtained from the model. The stress relaxation times, shapes and time scales over a broad range of temperatures and cross-link densities could be accurately predicted by the proposed model, based on the combination of the retro Diels–Alder reaction, the M–M network structure model, and an effectiveness factor, relying on only two temperature-independent parameters: an attenuation parameter *a* that depends on the type of network structure, and the activation parameter *b*, common in both networks. This unprecedented accuracy is achieved using minimal computational power.

Quantitative prediction of stress relaxation kinetics is possible if the bond exchange rate constants are known. In this sense, this modeling approach can also be used to derive the bond exchange kinetic parameters and related thermodynamic/equilibrium data from experimental stress relaxation experiments. These kinetic data could then be used to simulate other scenarios with the same or other dynamic networks. Moreover, even if bond exchange kinetics data are not available, this methodology facilitates the exploration of multiple composition/structure scenarios and the identification of kinetic trends that can be leveraged in application- and process-specific material design.

This intuitive description of the stress relaxation kinetics can constitute a module in a comprehensive model considering the following effects on the dynamic behavior: (i) the functionality and composition of the components in the network structure, (ii) the existence of permanent bonds or components with different bond exchange kinetics, (iii) the network mobility, (iv) the dynamic bond concentration, and (v) the presence of network defects, such as unreacted groups, loose chain ends, and intramolecular loops. This should help establish a comprehensive modeling framework for analyzing diverse scenarios and predicting trends and reprocessing capabilities across a broad range of CANs, prior to the experimental analysis and testing phase, without the need for going to computationally expensive modeling tools.

## Supplementary Material



## Notes

Complete kinetic-structural model for the cross-linking of reversible Diels–Alder networks; detailed analytical and Macosko–Miller kinetic-structural models for the stress relaxation; schemes of reactants; and additional model outputs and fitting results (tables and figures) (PDF)
