# Supplementary material for: Quantitative Prediction of Stress Relaxation Kinetics in Dissociative Covalent Adaptable Networks
Source: Macromolecules. 2025 Dec 3;58(24):13074–93. doi: 10.1021/acs.macromol.5c02070 (PMC12752708; doi:10.1021/acs.macromol.5c02070)
Supplement: Supplementary file 1 [file ma5c02070_si_001.pdf]

Quantitative prediction of stress relaxation kinetics in dissociative covalent adaptable networks

Jessica Mangialetto[1], Osman Konuray[2], Sasan Moradi[2], Xavier Fernández-Francos[2]*, Joost Brancart[1], Niko Van den Brande[1], Xavier Ramis[2], Guy Van Assche[1]

[1]Sustainable Materials Engineering, Vrije Universiteit Brussel (VUB), Pleinlaan 2, Brussels 1050, Belgium

[2]Thermodynamics Laboratory, ETSEIB, Universitat Politècnica de Catalunya, Av. Diagonal 647, 08028 Barcelona, Spain

Supporting information

1 Crosslinking kinetics 2

1.1 Kinetic model 2

1.2 Crosslinking process 5

2 Stress relaxation analysis 10

2.1 General considerations 10

2.2 Analytical model 12

2.3 Macosko-Miller model for stress relaxation 18

2.4 General model for stress-relaxation of Diels-Alder networks 19

3 Materials 23

4 Results 24

4.1 Analysis of model parameters 24

Analytical model 24

Effectiveness factor 25

4.2 Analysis of experimental results 26

5 References 34

1 Crosslinking kinetics

1.1 Kinetic model

The kinetic model is based on previous reaction mechanism of the furan-maleimide Diels-Alder reaction taking into consideration the existence of exo and endo adducts with different reaction kinetics and thermodynamical equilibrium.[1,2] If we call A the furan species, and B the maleimide species, we can write:

$$A + B \rightleftarrows AB_{endo}$$

$$A + B \rightleftarrows AB_{exo}$$

Where each equilibrium reaction is characterized by the rate constants of the forward and backward reactions, $k_{DA,xxx}$ and $k_{rDA,xxx}$ respectively, where xxx stands for *endo* or *exo* depending on the reaction under consideration.

The following rate expressions can be written for the reaction of the A and B species:

$$\frac{d[A]}{dt} = -\left(k_{DA,endo} + k_{DA,exo}\right) \cdot [A] \cdot [B] + k_{rDA,endo} \cdot [AB]_{endo} + k_{rDA,exo} \cdot [AB]_{exo}$$

$$\frac{d[B]}{dt} = \frac{d[A]}{dt}$$

And for the endo and exo adducts we can write:

$$\frac{d[AB]_{endo}}{dt} = k_{DA,endo} \cdot [A] \cdot [B] - k_{rDA,endo} \cdot [AB]_{endo}$$

$$\frac{d[AB]_{exo}}{dt} = k_{DA,exo} \cdot [A] \cdot [B] - k_{rDA,exo} \cdot [AB]_{exo}$$

These reactions fulfill this mass balance:

$$[A] + [B] + [AB]_{endo} + [AB]_{exo} = [A]_0 + [B]_0$$

For a stoichiometric system, the initial concentration of furan and maleimide groups are , $[A]_0 = [B]_0$. In consequence:

$$[A]_0 + [B]_0 = 2 \cdot [A]_0$$

We can define the concentration of the different species depending on the conversion of the endo and exo reactions as:

$$[AB]_{endo} = [A]_0 \cdot x_{endo}$$

$$[AB]_{exo} = [A]_0 \cdot x_{exo}$$

$$x = x_{endo} + x_{exo}$$

$$[A] = [A]_0 \cdot (1 - x_{endo} - x_{exo}) = [A]_0 \cdot (1 - x) = [B]$$

In consequence, we can rewrite the rate expressions as:

$$\frac{dx_{endo}}{dt} = k_{DA,endo} \cdot [A]_0 \cdot (1 - x)^2 - k_{rDA,endo} \cdot x_{endo}$$

$$\frac{dx_{exo}}{dt} = k_{DA,exo} \cdot [A]_0 \cdot (1 - x)^2 - k_{rDA,exo} \cdot x_{exo}$$

$$\frac{dx}{dt} = \left(k_{DA,endo} + k_{DA,exo}\right) \cdot [A]_0 \cdot (1 - x)^2 - k_{rDA,endo} \cdot x_{endo} + k_{rDA,exo} \cdot x_{exo}$$

This is further simplified to:

$$\frac{dx}{dt} = \left(k'_{DA,endo} + k'_{DA,exo}\right) \cdot (1 - x)^2 - k'_{rDA,endo} \cdot x_{endo} + k'_{rDA,exo} \cdot x_{exo}$$

With rate constants:

$$k'_{DA,endo} = k_{DA,endo} \cdot [A]_0 \qquad k'_{rDA,endo} = k_{rDA,endo}$$

$$k'_{DA,exo} = k_{DA,exo} \cdot [A]_0 \qquad k'_{rDA,exo} = k_{rDA,exo}$$

The rate constants are calculated from the following expressions

$$k_{DA,endo} = \exp\left(\ln k_{0,DA,endo} - \frac{E_{DA,endo}}{R \cdot T}\right)$$

$$k_{rDA,endo} = \exp\left(\ln k_{0,rDA,endo} - \frac{E_{rDA,endo}}{R \cdot T}\right)$$

$$k_{DA,exo} = \exp\left(\ln k_{0,DA,exo} - \frac{E_{DA,exo}}{R \cdot T}\right)$$

$$k_{rDA,exo} = \exp\left(\ln k_{0,rDA,exo} - \frac{E_{rDA,exo}}{R \cdot T}\right)$$

The kinetic parameters to determine each constant are tabulated in Table S1.[1]

Table S - 1: Kinetic parameters of the forward and backward D-A reactions involving the *endo* and *exo* adducts.

**Endo reaction**		**Exo reaction**	
$\ln k_{0,DA,endo}$ $(kg \cdot mol^{-1} \cdot s^{-1})$	13.4	$\ln k_{0,DA,exo}$ $(kg \cdot mol^{-1} \cdot s^{-1})$	14.7
$E_{DA,endo}$ $(kJ \cdot mol^{-1})$	59.4	$E_{DA,exo}$ $(kJ \cdot mol^{-1})$	64.6
$\ln k_{0,rDA,endo}$ (s^{-1})	30.9	$\ln k_{0,rDA,exo}$ (s^{-1})	31.4
$E_{rDA,endo}$ $(kJ \cdot mol^{-1})$	113.1	$E_{rDA,exo}$ $(kJ \cdot mol^{-1})$	123.6

For a given temperature programme, these rate equations can be integrated numerically using any convenient method. An equilibrium conversion x_{eq} will eventually be reached depending on the temperature. This equilibrium can be defined from the rate expressions, by stating $dx_{exo}/dt = 0$ and $dx_{endo}/dt = 0$

$$k'_{DA,exo} \cdot \left(1 - x_{eq}\right)^2 = k'_{rDA,exo} \cdot x_{exo,eq} \Rightarrow x_{exo,eq} = \frac{k'_{DA,exo} \cdot \left(1 - x_{eq}\right)^2}{k'_{rDA,exo}}$$

$$k'_{DA,endo} \cdot \left(1 - x_{eq}\right)^2 = k'_{rDA,endo} \cdot x_{endo,eq} \Rightarrow x_{endo,eq} = \frac{k'_{DA,endo} \cdot \left(1 - x_{eq}\right)^2}{k'_{rDA,endo}}$$

Given that $x_{eq} = x_{exo,eq} + x_{endo,eq}$, we can write

$$x_{eq} = \left(\frac{k'_{DA,exo}\cdot}{k'_{rDA,exo}} + \frac{k'_{DA,endo}}{k'_{rDA,endo}}\right)\cdot\left(1 - x_{eq}\right)^2$$

A global equilibrium can be defined:

$$K'_{eq} = \frac{x_{eq}}{\left(1 - x_{eq}\right)^2} = \frac{k'_{DA,exo}}{k'_{rDA,exo}} + \frac{k'_{DA,endo}}{k'_{rDA,endo}}$$

$$K'_{eq}\cdot x^2 - \left(1 + 2\cdot K'_{eq}\right)\cdot x + K'_{eq} = 0$$

$$x_{eq} = \frac{\left(1 + 2\cdot K'_{eq}\right) - \sqrt{\left(1 + 2\cdot K'_{eq}\right)^2 - 4\cdot K'_{eq}{}^2}}{2\cdot K'_{eq}} = \frac{\left(1 + 2\cdot K'_{eq}\right) - \sqrt{1 + 4\cdot K'_{eq}}}{2\cdot K'_{eq}}$$

With the values of x_{eq}, we can also calculate equilibrium values of x_{exo} and x_{endo}.

1.2 Crosslinking process

Let us consider the initial concentration of furan and maleimide species $\left[A_f\right]_0$ and $\left[B_g\right]_0$, with functionality f and g respectively:

$$[A]_0 = \left[A_f\right]_0\cdot f = \left[B_g\right]_0\cdot g = [B]_0$$

A distribution of species with different numbers of reacted bonds can be determined depending on the global extent of DA reaction, calculated using the kinetic model in the preceding section. These reacted species constitute structural fragments containing bonds that are connected with each other following certain rules. The A_f component will lead to a distribution of $A_{f,n}$ fragments with n reacted bonds labeled with (+), and the B_g component will lead to a distribution of $B_{g,n}$ fragments with n reacted bonds labeled with (-). A probable polymer/network structure is formed by the random combination of (+) and (-) bonds from the different structural fragments.

The distribution of $A_{f,n}$ fragments is given by:

$$\left[A_{f,n}\right] = \left[A_f\right]_0 \cdot \binom{f}{n} \cdot (1-x)^{f-n} \cdot x^n$$

Considering the initial distribution of monomers, the total concentration of reacted A groups and therefore (+) bonds is given by

$$[+] = [A]_0 \cdot x = \left[A_f\right]_0 \cdot f \cdot x = \sum_{n=0}^{f} \left(\left[A_{f,n}\right] \cdot n\right) = \left[A_f\right]_0 \cdot \sum_{n=0}^{f} \left(n \cdot \binom{f}{n} \cdot (1-x)^{f-n} \cdot x^n\right)$$

Similarly, for the $B_{g,n}$ species:

$$\left[B_{g,n}\right] = \left[B_g\right]_0 \cdot \binom{g}{n} \cdot (1-x)^{g-n} \cdot x^n$$

Also:

$$[-] = [B]_0 \cdot x = \left[B_g\right]_0 \cdot g \cdot x = \sum_{n=0}^{g} \left(\left[B_{g,n}\right] \cdot n\right) = \left[B_g\right]_0 \cdot \sum_{n=0}^{g} \left(n \cdot \binom{g}{n} \cdot (1-x)^{g-n} \cdot x^n\right)$$

The total number of connecting (+) and (-) bonds must be equal, $[+] = [-]$

We can define the capture probabilities of the different fragments with (+) bonds as:

$$P^{+}_{A_{f,n}} = \frac{n \cdot \left[A_{f,n}\right]}{[+]} = \frac{n \cdot \left[A_f\right]_0 \cdot \binom{f}{n} \cdot (1-x)^{f-n} \cdot x^n}{\left[A_f\right]_0 \cdot f \cdot x} = \frac{n \cdot \binom{f}{n} \cdot (1-x)^{f-n} \cdot x^n}{f \cdot x}$$

For the fragments with (-) bonds:

$$P^{-}_{B_{g,n}} = \frac{n \cdot \left[B_{g,n}\right]}{[-]} = \dots = \frac{n \cdot \binom{g}{n} \cdot (1-x)^{g-n} \cdot x^n}{g \cdot x}$$

For the pregel state, we can define the *expected weights* W^{+} and W^{-} pending from the (+) and (-) bonds, which are calculated recursively from the probability of capturing a fragment with complementary bond and the attached mass, which includes the mass of that fragment, and the expected weight pending from the remaining bonds, as:

$$W^{+} = \sum_{n=1}^{g} \left(P_{B_{g,n}}^{-} \cdot \left(M_{B_{g,n}} + (n-1) \cdot W^{-} \right) \right)$$

$$W^{-} = \sum_{n=1}^{f} \left(P_{A_{f,n}}^{+} \cdot \left(M_{A_{f,n}} + (n-1) \cdot W^{+} \right) \right)$$

Where $M_{A_{f,n}}$ and $M_{B_{g,n}}$ are the molar masses of fragments $A_{f,n}$ and $B_{g,n}$ respectively, which can be made equal to the molar mass of starting components A_f and B_g, M_{A_f} and M_{B_g}.

This can be expressed in matrix form, $\boldsymbol{M} \cdot \boldsymbol{W} = \boldsymbol{I}$, as:

$$\begin{pmatrix} 1 & m_{+,-} \\ m_{-,+} & 1 \end{pmatrix} \cdot \begin{pmatrix} W^{+} \\ W^{-} \end{pmatrix} = \begin{pmatrix} I^{+} \\ I^{-} \end{pmatrix}$$

Where the coefficients of the matrix $\boldsymbol{M}$ are equal to

$$m_{+,-} = -\sum_{n=1}^{g} \left(P_{B_{g,n}}^{-} \cdot (n-1) \right)$$

$$m_{-,+} = -\sum_{n=1}^{f} \left(P_{A_{f,n}}^{+} \cdot (n-1) \right)$$

And the independent terms can be calculated as:

$$I^{+} = \sum_{n=1}^{g} \left(P_{B_{g,n}}^{-} \cdot M_{B_{g,n}} \right) = M_{B_g}$$

$$I^{-} = \dots = M_{A_f}$$

Before gelation, $\det \boldsymbol{M} > 0$, which leads to positive and finite solutions of W^{+} and W^{-} and a probable distribution of polymer species with a finite mass-average molecular weight M_w that can be calculated as

$$M_w = \sum_{n=1}^{f} \left(w_{A_{f,n}} \cdot \left(M_{A_{f,n}} + n \cdot W^+ \right) \right) + \sum_{n=1}^{g} \left(w_{B_{g,n}} \cdot \left(M_{B_{g,n}} + n \cdot W^- \right) \right)$$

With

$$w_{A_{f,n}} = \frac{\left[A_{f,n}\right] \cdot M_{A_{f,n}}}{\sum\left[A_{f,n}\right] \cdot M_{A_{f,n}} + \sum\left[B_{g,n}\right] \cdot M_{B_{g,n}}}$$

$$w_{B_{g,n}} = \frac{\left[B_{g,n}\right] \cdot M_{B_{g,n}}}{\sum\left[A_{f,n}\right] \cdot M_{A_{f,n}} + \sum\left[B_{g,n}\right] \cdot M_{B_{g,n}}}$$

Gelation is given by condition that the *expected weights* W^+ and W^- become infinite, leading to $M_w \longrightarrow \infty$. This is determined from the condition that $\det \boldsymbol{M} = 0$, which leads to:

$$m_{+,-} \cdot m_{-,+} = 1$$

Which is equivalent to

$$x_{gel} = \sqrt{\frac{1}{(f-1) \cdot (g-1)}}$$

Please notice that this expression can be generalized to distributions of A and B components[3,4], with:

$$x_{gel} = \sqrt{\frac{1}{(f" - 1) \cdot (g" - 1)}}$$

Where $f"$ and $g"$ are the second moment of the monomer functionalities, defined as:

$$f" = \frac{\Sigma_f\left(\left[A_f\right]_0 \cdot f^2\right)}{\Sigma_f\left(\left[A_f\right]_0 \cdot f\right)} \qquad g" = \frac{\Sigma_g\left(\left[B_g\right]_0 \cdot g^2\right)}{\Sigma_g\left(\left[B_g\right]_0 \cdot g\right)}$$

For the postgel state, we define the *extinction probabilities* Z^+ and Z^- of the (+) and (-) bonds, which represent the likelihood of finding a finite branch when looking outwards from a given (+)

or (-) bond respectively. These probabilities are calculated recursively from the probability of capturing a fragment with complementary bond and the probability that the captured fragment has a finite continuation, taking into consideration the remaining bonds, as:

$$Z^{+} = \sum_{n=1}^{g} \left(P^{-}_{B_{g,n}} \cdot (Z^{-})^{n-1} \right)$$

$$Z^{-} = \sum_{n=1}^{f} \left(P^{+}_{A_{f,n}} \cdot (Z^{+})^{n-1} \right)$$

The number of elastically active network strands (EANS) is defined from the structural fragments containing at least 3 bonds to the network (infinite continuation) and the application of the phantom network model as:

$$n_{EANS} = \sum_{n=3}^{f} \left(\left[A_{f,n}\right] \cdot \sum_{a=3}^{n} \left(\left(\frac{a}{2} - 1\right) \cdot \binom{n}{a} \cdot (1 - Z^{+})^{a} \cdot (Z^{+})^{n-a} \right) \right) + \sum_{n=3}^{g} \left(\left[B_{g,n}\right] \cdot \sum_{a=3}^{n} \left(\left(\frac{a}{2} - 1\right) \cdot \binom{n}{a} \cdot (1 - Z^{-})^{a} \cdot (Z^{-})^{n-a} \right) \right)$$

The total number of network strands is given by:

$$n_{strand} = \sum_{n=3}^{f} \left(\left[A_{f,n}\right] \cdot \sum_{a=3}^{n} \left(\left(\frac{a}{2}\right) \cdot \binom{n}{a} \cdot (1 - Z^{+})^{a} \cdot (Z^{+})^{n-a} \right) \right) + \sum_{n=3}^{g} \left(\left[B_{g,n}\right] \cdot \sum_{a=3}^{n} \left(\left(\frac{a}{2}\right) \cdot \binom{n}{a} \cdot (1 - Z^{-})^{a} \cdot (Z^{-})^{n-a} \right) \right)$$

The fraction of EANS with respect to the total network strands f_{EANS} is given by:

$$f_{EANS} = \frac{n_{EANS}}{n_{strand}}$$

The total number of (+) network bonds is determined from the fragments containing at least 2 bonds to the network structure as:

$$n_{(+),net} = \sum_{n=2}^{f}\left(\left[A_{f,n}\right]\cdot \sum_{a=2}^{n}\left(a\cdot \binom{n}{a}\cdot(1-Z^{+})^{a}\cdot(Z^{+})^{n-a}\right)\right)$$

The total number of (-) network bonds:

$$n_{(-),net} = \sum_{n=2}^{g}\left(\left[B_{g,n}\right]\cdot \sum_{a=2}^{n}\left(a\cdot \binom{n}{a}\cdot(1-Z^{-})^{a}\cdot(Z^{-})^{n-a}\right)\right)$$

It is also necessary that $n_{(+),net} = n_{(-),net}$

The total number of (+) or (-) bonds in the EANS (not the total network strands) is given by:

$$n_{(+),EANS} = f_{EANS}\cdot n_{(+),net} \qquad n_{(-),EANS} = f_{EANS}\cdot n_{(-),net}$$

$$n_{(+),EANS} = n_{(-),EANS}$$

2 Stress relaxation analysis

2.1 General considerations

Let us consider the bond exchange process leading to the relaxation of stress. At the beginning of the process, we can label all the dynamic bonds D as non-exchanged bonds, having a concentration $[D]_{n-e}$. As the bond exchange advances, these dynamic bonds are exchanged and transformed into exchanged bonds, with a concentration $[D]_{e}$:

$$[D]_{n-e} \xrightarrow{exchange} [D]_{e}$$

These exchanged bonds can continue participating in bond exchange events, but their status of exchanged bonds is not affected:

$$[D]_{e} \xrightarrow{exchange} [D]_{e}$$

From a kinetics perspective, this can be expressed as:

$$\frac{d[D]_{n-e}}{dt} = - k_{xch} \cdot [D]_{n-e}$$

$$\frac{d[D]_{e}}{dt} = k_{xch} \cdot [D]_{n-e}$$

Where k_{xch} is the apparent bond exchange rate constant, which depends obviously temperature but could also depend on the total concentration of dynamic bonds, catalytic species, or the mobility of the network structure, depending on the type of bond exchange mechanism.

This set of reactions leads to the obvious consequence that the total number of bonds remain constant:

$$[D]_{n-e} + [D]_{e} = [D]_{total} = ct$$

$$\frac{d[D]_{n-e}}{dt} + \frac{d[D]_{e}}{dt} = 0$$

It was recently proposed that the stress relaxation process could be likened to a network decrosslinking process, in which the exchange of bonds leads to a deactivation of the elastic activity of the network strands, which is similar to the cleavage of these dynamic bonds. Making use of this analogy and of a suitable network build-up model (a mean-field model based on the Macosko-Miller recursive approach[3,5]), it was recently shown that it was not necessary to exchange all the bonds in the network structure in order to achieve complete stress relaxation.[5] The model enabled the prediction of the effect of non-dynamic bonds in the network structure and the existence of complex stress relaxation profiles due to the combination of bond exchange reactions with different kinetics.

The mean-field model represents the effect of the random exchange bonds in the network structure. However, it was also acknowledged that only the exchange of bonds taking place in the network strands with remaining elastic activity would have an impact.

In consequence, we will first analyze the effect of an analytical model, based on the analysis of the remaining network in the network structure, and compare it with the output from the Macosko-Miller approach.

2.2 Analytical model

A fully crosslinked $A_4 - B_2$ network is considered. This implies that, at the beginning, the system is composed of $A_{4,net}$ fragments containing 4 $(+)_{net}$ bonds, and $B_{2,net}$ fragments containing 2 $(-)_{net}$bonds that are randomly connected with each other. The basic assumption of this model is that a bond exchange event in a network strand will deactivate the whole network strand, and further bond exchange events in the affected fraction of material will produce no further effect.

This process can be likened to a network decrosslinking process in which the affected network strands are removed from the system, so that no dangling chains nor soluble fraction is present in the active network, and only a network structure with decreasing functionality and density remains.

This implies that, upon a bond exchange event, the affected crosslink junctions at both ends of the network strand will decrease functionality (and this will imply a transformation from $A_{4,net}$ species to $A_{3,net}$) or else will turn into linear units (e.g. from $A_{3,net}$ to $A_{2,net}$). At the same time, linear units in that network strand ($A_{2,net}$ and $B_{2,net}$) will disappear.

In consequence, we will have a system in which only the following fragments will be present:

- $A_{4,net}$: tetrafunctional crosslink with 4 $(+)_{net}$ bonds
- $A_{3,net}$: trifunctional crosslink with 3 $(+)_{net}$ bonds
- $A_{2,net}$: difunctional linear unit with 2 $(+)_{net}$ bonds
- $B_{2,net}$: difunctional linear unit with 2 $(-)_{net}$ bonds

In the process, the number of $(+)_{net}$ and $(-)_{net}$ bonds must always be equal

$$n_{(+),net} = 4 \cdot [A_{4,net}] + 3 \cdot [A_{3,net}] + 2 \cdot [A_{2,net}] = 2 \cdot [B_{2,net}] = n_{(-),net}$$

It should be noticed that, according to this model, the existing (+) and (-) bonds are bonds that belong to the active network structure, so that there are not dangling chains nor soluble fractions of material.

At the beginning of the process there are only A_4 and B_2 units, so that

$$n_{(+),net} = 4 \cdot [A_{4,net}]_0 = 2 \cdot [B_{2,net}]_0 = n_{(-),net}$$

$$[A_{3,net}]_0 = 0 \qquad [A_{2,net}]_0 = 0$$

The kinetic model can be described starting from a basic rate expression that describes the rate of disappearance of a network strand upon a bond exchange event in that strand, which depends on the number of $(+)_{net}$ or $(-)_{net}$ bonds in the network structure, as:

$$\frac{d[strand]}{dt} = -k_{xch} \cdot n_{(-),net} = -k_{xch} \cdot 2 \cdot [B_{2,net}] = -r_{xch}$$

Now let us write the rate expressions for all individual species. The $A_{4,net}$ species will disappear depending on the probability that $A_{4,net}$ is found at the ends of the strand affected by the bond exchange event:

$$\frac{d[A_{4,net}]}{dt} = -r_{xch} \cdot 2 \cdot f_{A4}$$

Where the factor 2 indicates that for each bond exchange event two chain-ends, crosslinking species (either $A_{4,net}$ or $A_{3,net}$) will be downgraded, and f_{A4} is the probability that the chain-end is $A_{4,net}$.

The $A_{3,net}$ species will appear as a consequence of the bond exchange events affecting $A_{4,net}$ (which are transformed into $A_{3,net}$) but will disappear when a bond exchange takes place in a network strand with A_3 at the ends of the strand:

$$\frac{d[A_{3,net}]}{dt} = r_{xch} \cdot 2 \cdot f_{A4} - r_{xch} \cdot 2 \cdot f_{A3} = -\frac{d[A_{4,net}]}{dt} - r_{xch} \cdot 2 \cdot f_{A3}$$

Where f_{A3} is the probability that $A_{3,net}$ is found as a chain-end in a network strand, and the factor 2 has the same meaning as before.

The $A_{2,net}$ species will appear as a consequence of the bond exchange events affecting $A_{3,net}$ (which are transformed into linear $A_{2,net}$) but will disappear upon deactivation of the network strand upon a bond exchange event, depending on the average number of $A_{2,net}$ units in the network strand:

$$\frac{d[A_{2,net}]}{dt} = r_{xch} \cdot 2 \cdot f_{A3,net} - r_{xch} \cdot L''_{A2} = -\frac{d[A_{3,net}]}{dt} - r_{xch} \cdot L''_{A2}$$

Where L''_{A2} is the average number of $A_{2,net}$ units in the affected network strand.

Finally, for $B_{2,net}$ we can write the following rate equation, describing the disappearance of bond exchange events in the network strand affected by the bond exchange event.

$$\frac{d[B_{2,net}]}{dt} = - r_{xch} \cdot L''_{B2}$$

Where L''_{B2} is the average number of $B_{2,net}$ units in the affected network strand.

The f_{A4} and f_{A3} factors are calculated depending on the contribution of $A_{4,net}$ and $A_{3,net}$ to the network strand ends, as:

$$f_{A4} = \frac{4 \cdot [A_{4,net}]}{3 \cdot [A_{3,net}] + 4 \cdot [A_{4,net}]} \qquad f_{A3} = 1 - f_{A4}$$

The rate expressions fulfill the condition that

$$\frac{dn_{(+),net}}{dt} = \frac{dn_{(-),net}}{dt}$$

$$4 \cdot \frac{d[A_{4,net}]}{dt} + 3 \cdot \frac{d[A_{3,net}]}{dt} + 2 \cdot \frac{d[A_{2,net}]}{dt} = 2 \cdot \frac{d[B_{2,net}]}{dt}$$

This can be verified as follows:

$$4 \cdot \frac{d[A_{4,net}]}{dt} = -4 \cdot r_{xch} \cdot 2 \cdot f_{A4}$$

$$3\cdot\frac{d[A_{3,net}]}{dt} = 3\cdot r_{xch}\cdot 2\cdot f_{A4} - 3\cdot r_{xch}\cdot 2\cdot f_{A3}$$

$$2\cdot\frac{d[A_{2,net}]}{dt} = 2\cdot r_{xch}\cdot 2\cdot f_{A3} - 2\cdot r_{xch}\cdot L"_{A2}$$

$$4\cdot\frac{d[A_{4,net}]}{dt} + 3\cdot\frac{d[A_{3,net}]}{dt} + 2\cdot\frac{d[A_{2,net}]}{dt} = -\, r_{xch}\cdot 2\cdot f_{A4} - r_{xch}\cdot 2\cdot f_{A3} - 2\cdot r_{xch}\cdot L"_{A2}$$

$$= -\, r_{xch}\cdot 2\cdot (f_{A4} + f_{A3} + L"_{A2}) = -2\cdot r_{xch}\cdot (1 + L"_{A2})$$

On the other hand:

$$2\cdot\frac{d[B_{2,net}]}{dt} = -2\cdot r_{xch}\cdot L"_{B2}$$

Given that in a network strand there should always be one more $B_{2,net}$ unit than $A_{2,net}$ unit (the crosslinks are always $A_{3,net}$ and $A_{4,net}$), that is, $L"_{B2} = L"_{A2} + 1$, the condition is fulfilled.

In order to determine correctly the parameters $L"_{A2}$ and $L"_{B2}$ we should compute correctly the average number of units that would be affected by a bond exchange.

Let us consider a network strand with length L_2, where L_2 is the total number of linear units in the chain, so that $L_{A2} + L_{B2} = L_2$ (equivalently, $L_2 = 2\cdot L_{B2} - 1$). The probability that a given chain is captured for a bond exchange depends on the number of B_2 units in the strand as:

$$P^i_{xch} = \frac{n_i\cdot L_{B2,i}}{\sum n_i\cdot L_{B2,i}}$$

Where n_i is the number of network strands with a number of B_2 units equal to $L_{B2,i}$. Now, we consider the number of B_2 units that would be lost providing a bond exchange event took place in that strand:

$$P^i_{xch}\cdot L_{B2,i} = \frac{n_i\cdot L_{B2,i}{}^2}{\sum n_i\cdot L_{B2,i}}$$

Now, if we want to compute the average number of B_2 units that would be lost:

$$L_{ave} = L"_{B2} = \sum P^{i}_{xch} \cdot L_{B2,i} = \frac{\sum n_i \cdot L_{B2,i}{}^2}{\sum n_i \cdot L_{B2,i}}$$

That is, the second moment of the number of B_2 units in a network strand.

To determine this, we will apply a recursive method of Macosko-Miller to determine the total number of segments in a network strand. This includes difunctional $A_{2,net}$ and $B_{2,net}$ units, and chain ends CE, which will correspond to the branches of $A_{4,net}$ or $A_{3,net}$ units with $(+)_{net}$ bonds. We will define the capture probabilities:

$$n_{(+),net} = 4 \cdot [A_{4,net}] + 3 \cdot [A_{3,net}] + 2 \cdot [A_{2,net}] = [CE] + 2 \cdot [A_{2,net}]$$

$$P^{+}_{CE} = \frac{CE}{n_{(+),net}} \qquad P^{+}_{A2} = \frac{2 \cdot [A_{2,net}]}{n_{(+),net}} = 1 - P^{+}_{CE} \qquad P^{-}_{B2} = \frac{2 \cdot [B_{2,net}]}{n_{(-),net}} = 1$$

The expected number of segments in network strands looking from (+) and (-) bonds can be calculated recursively as:

$$N^{+} = P^{-}_{B2} \cdot (1 + N^{-}) = 1 + N^{-}$$

$$N^{-} = P^{+}_{A2} \cdot (1 + N^{+}) + P^{+}_{CE} = P^{+}_{A2} \cdot (1 + N^{+}) + 1 - P^{+}_{A2} = 1 + P^{+}_{A2} \cdot N^{+}$$

Please note that linear units contribute with one segment plus additional segments pending from remaining bond, while chain ends only contribute with one segment.

Solving leads to:

$$N^{+} = 2 + P^{+}_{A2} \cdot N^{+}$$

$$N^{+} = \frac{2}{1 - P^{+}_{A2}} \qquad N^{-} = N^{+} - 1 = \frac{2}{1 - P^{+}_{A2}} - 1 = \frac{1 + P^{+}_{A2}}{1 - P^{+}_{A2}}$$

Now we calculate the second moment of the number of segments in a polymer chain, which depends on the difunctional units in the chains (contributing with 1 segment and $2 \cdot N^{+}$ or $2 \cdot N^{-}$) and the chain ends (contributing with 1 segment and N^{+}):

$$N" = \frac{[CE]\cdot(1 + N^{+}) + [A_{2,net}]\cdot(1 + 2\cdot N^{+}) + [B_{2,net}]\cdot(1 + 2\cdot N^{-})}{[CE] + [A_{2,net}] + [B_{2,net}]}$$

Then, it must be acknowledged that the average number of difunctional units is equal to the total number of segments minus 2 chain ends:

$$L"=N" - 2 = \dots = \frac{[CE]\cdot(N^{+} - 1) + [A_{2,net}]\cdot(2\cdot N^{+} - 1) + [B_{2,net}]\cdot(2\cdot N^{-} - 1)}{[CE] + [A_{2,net}] + [B_{2,net}]}$$

Finally, the contribution of $B_{2,net}$ and $A_{2.net}$ units is given by:

$$L"_{B2} = \frac{L" + 1}{2} \qquad L"_{A2} = \frac{L" - 1}{2}$$

These expressions are incorporated into the kinetic model. The set of rate expressions are integrated numerically using a convenient method.

The evolution of the remaining stress is calculated from the fraction of elastically active network strands (EANS), using the phantom network model, as:

$$n_{EANS} = \left(\frac{4}{2} - 1\right)\cdot[A_{4,net}] + \left(\frac{3}{2} - 1\right)\cdot[A_{3,net}]$$

$$f_{stress} = \frac{n_{EANS}}{n_{EANS,t=0}}$$

The number of bond exchange events leading to stress relaxation can be computed in the numerical integration process from:

$$n_{xch,relax} = \int r_{xch}\cdot dt$$

The fraction of bond exchange events with respect to the total initial number of dynamic bonds can be calculated as:

$$f_{xch} = \frac{n_{xch,relax}}{2\cdot[B_{2,net}]_0}$$

Please note that a very similar model could be defined for an $A_3 - B_2$ system. The same system of equations can be used in fact, but taking as starting values:

$$n_{(+),net} = 3 \cdot [A_{3,net}]_0 = 2 \cdot [B_{2,net}]_0 = n_{(-),net}$$

$$[A_{4,net}]_0 = 0 \qquad [A_{2,net}]_0 = 0$$

The fraction of EANS with respect to the total number of dynamic bonds present in the system at the beginning of the relaxation process is given by:

$$f_{EANS,bond} = \frac{n_{EANS}}{2 \cdot [B_{2,net}]_0} = \frac{\left(\frac{4}{2} - 1\right) \cdot [A_{4,net}]_0 + \left(\frac{3}{2} - 1\right) \cdot [A_{3,net}]_0}{2 \cdot [B_{2,net}]_0}$$

For an $A_4 - B_2$ system (no trifunctional units originally present in the network structure), $f_{EANS,bond} = 0.25$. For an $A_3 - B_2$ system, $f_{EANS,bond} = 0.17$.

2.3 Macosko-Miller model for stress relaxation

The general relaxation model makes use of the general Macosko-Miller model used for the crosslinking process (all expressions available in section 1.2), but applied to a decrosslinking process. The general rate expression for the stress relaxation process can be expressed as follows:

$$\frac{d[AB]}{dt} = -\, k_{xch} \cdot [AB] \qquad \frac{dx}{dt} = -\, k_{xch} \cdot x$$

Where k_{xch} is the rate constant of the bond exchange process, and x reflects the extent of bond formation. Given that the stress relaxation process is assimilated to a decrosslinking process in which bond cleavage is equivalent to bond exchange, the value of x should decrease in the process.

These expressions are integrated using a convenient numerical method. The general algorithm is as follows:

1. For a given value of x, we calculate
    - the distribution of connected species $A_{f,n}$ and $B_{g,n}$ depending on x (section 1.2)

- the capture probabilities $P^{+}_{A_{f,n}}$ and $P^{-}_{B_{g,n}}$ (section 1.2)
- the matrix M and $\det M$ (stop here if $\det M > 0$)
- the extinction probabilities, $Z_{(+)}$ and $Z_{(-)}$ (section 1.2)
- the number of EANS n_{EANS} (section 1.2)
- the residual stress $f_{stress} = n_{EANS}/n_{EANS,t=0}$ (stop here if $f_{stress} < \varepsilon$, with ε a defined tolerance).

2. For a given timestep Δt, we compute $\Delta x = -k_{xch}{\cdot}x{\cdot}\Delta t = -x{\cdot}(1-\exp(-k_{xch}{\cdot}\Delta t))$
3. The value of x is updated $x' = x + \Delta x$, then $x = x'$.
4. The value of t is updated, $t' = t + \Delta t$, then $t = t'$.
5. Loop back to step 1.

The algorithm starts at $t = 0$ and $x = x_0$, where $x_0 = 1$ for a fully crosslinked network or $x_0 = x_{eq}$ for an equilibrium Diels-Alder network (or other dissociative CANs). For the sake of computational economy, an adaptive algorithm was employed such that Δt is adjusted upwards after each iteration respecting a predefined error tolerance.

According to the decrosslinking analogy, the stress relaxation process should end when degelation is reached, that is, when $x = x_{gel}$, leading to $n_{(+),EANS} = 0$. For an ideal, stoichiometric step-growth polymerization network, this value of x_{gel} can be easily determined as:

$$x_{gel} = \sqrt{\frac{1}{(f_A - 1){\cdot}(f_B - 1)}}$$

Where f_A and f_B are the functionalities of the components in the network structure.

2.4 General model for stress-relaxation of Diels-Alder networks

The general model for the analysis of a stress relaxation process takes into consideration that the effectiveness of bond exchange events taking place in the material is not the same throughout the entire stress relaxation process. In consequence, the general rate expression for the stress relaxation process can be expressed as follows:

$$\frac{d[AB]}{dt} = -f_{eff} \cdot k_{xch} \cdot [AB] \qquad \frac{dx}{dt} = -f_{eff} \cdot k_{xch} \cdot x$$

In previous works, this effectiveness factor has been defined by assuming that the deactivation of polymer chains containing non-exchanged bonds, leads to a decrease in the effectiveness of bond exchange events.[5] However, only approximate definitions of f_{eff} were given, which made it possible to produce stress relaxation curves with appropriate shape.

However, in the present work we choose to elaborate a more realistic definition of f_{eff}. The starting hypothesis is that kinetic rate constants may be affected by the existence of a strain-induced stress disturbing the energy level of the dynamic bonds in network strands under load, because of a change the conformation of polymer chains to a less stable one. This perturbation would decrease the activation energy of the bond exchange process for that given bond in the network strain under load:

$$\Delta E = b$$

$$E'_{xch} = E_{xch} - \Delta E$$

It is hypothesized that such perturbation might not depend on the level of strain: increasing strain may change the global configuration of polymer chains, but the change in the local energetic perturbation of the dynamic bond will not be proportional to the strain.

If we consider the global system, we can assume that only a fraction of the dynamic bonds in the network structure participates in the mechanical response of the material, therefore the global effect would be proportional to the amount of remaining AB groups under load, that is, belonging to the EANS, $[AB]_{EANS}$, with respect to the total number of AB groups in the material, $[AB]_{total}$, which remains constant throughout the relaxation process. Therefore, we can write:

$$\Delta E = \frac{[AB]_{EANS}}{[AB]_{total}} \cdot b$$

In the expression, $[AB]_{EANS}$ should be equal to the number of (+) or (-) bonds in the EANS, that is, $[AB]_{EANS} = n_{(+),EANS} = n_{(-),EANS}$ (see section 1.2 for their formal definition). With regards

to the total number of AB groups, we consider the maximum possible amount if the network was fully crosslinked $[AB]_{total} = [A]_0$. This leads to:

$$\Delta E = f_{(+),EANS} \cdot b \qquad f_{(+),EANS} = \frac{n_{(+),EANS}}{[A]_0}$$

Where $f_{(+),EANS}$ is the fraction of dynamic bonds under stress. In consequence, the activation energy of the bond exchange process is modified as follows:

$$E'_{xch} = E_{xch} - f_{(+),EANS} \cdot b$$

And the rate constant would be affected as follows:

$$k'_{xch} = k_0 \cdot \exp\left(-\frac{E'_{xch}}{R \cdot T}\right) = k_0 \cdot \exp\left(-\frac{E_{xch}}{R \cdot T} + \frac{f_{(+),EANS} \cdot b}{R \cdot T}\right)$$

$$= \exp\left(\frac{f_{(+),EANS} \cdot b}{R \cdot T}\right) \cdot k_0 \cdot \exp\left(-\frac{E_{xch}}{R \cdot T}\right) = f_{eff} \cdot k_{xch}$$

This justifies the existence of an effectiveness factor depending on the state of stress relaxation with respect to the equilibrium network, which is also temperature-dependent.

$$f_{eff} = \exp\left(\frac{f_{(+),EANS} \cdot b}{R \cdot T}\right)$$

This effectiveness factor takes values which are higher than 1 at the beginning of the relaxation process, decreasing to a value of 1 when the relaxation process is over and $n_{(+),EANS} = 0$.

Assuming that, for a dissociative Diels-Alder network, the rate bond exchange process is equivalent to the rate of the dissociation reaction, and the existence of both *endo* and *exo* adducts, the kinetic-structural model can be redefined starting from the following rate expressions:

$$\frac{dx_{endo}}{dt} = -f_{eff} \cdot k'_{rDA,endo} \cdot x_{endo}$$

$$\frac{dx_{exo}}{dt} = -f_{eff} \cdot k'_{rDA,exo} \cdot x_{exo}$$

$$\frac{dx}{dt} = -f_{eff} \cdot \left(k'_{rDA,endo} \cdot x_{endo} + k'_{rDA,exo} \cdot x_{exo}\right)$$

These expressions are integrated using a convenient numerical method, following a similar algorithm as in the simple Macosko-Miller method:

1. For a given value of $x = x_{endo} + x_{exo}$, we calculate
   - the distribution of connected species $A_{f,n}$ and $B_{g,n}$ depending on x (section 1.2)
   - the capture probabilities $P^{+}_{A_{f,n}}$ and $P^{-}_{B_{g,n}}$ (section 1.2)
   - the matrix M and $\det M$ (stop here if $\det M > 0$)
   - the extinction probabilities, $Z_{(+)}$ and $Z_{(-)}$ (section 1.2)
   - the number of EANS n_{EANS} (section 1.2)
   - the residual stress $f_{stress} = n_{EANS}/n_{EANS,t=0}$ (stop here if $f_{stress} < \varepsilon$, with ε a defined tolerance)
   - the effectiveness factor f_{eff} as defined in this section.
2. For a given timestep Δt, we compute the change in the different adducts:
   - $\Delta x_{endo} = -f_{eff} \cdot k'_{rDA,endo} \cdot x_{endo} \cdot \Delta t = -x_{endo} \cdot \left(1 - \exp\left(-f_{eff} \cdot k'_{rDA,endo} \cdot \Delta t\right)\right)$
   - $\Delta x_{exo} = -f_{eff} \cdot k'_{rDA,exo} \cdot x_{exo} \cdot \Delta t = -x_{exo} \cdot \left(1 - \exp\left(-f_{eff} \cdot k'_{rDA,exo} \cdot \Delta t\right)\right)$
   - $\Delta x = \Delta x_{endo} + \Delta x_{exo}$
3. The value of x is updated $x_{endo}' = x_{endo} + \Delta x_{endo}$, $x_{exo}' = x_{exo} + \Delta x_{exo}$, $x' = x + \Delta x$ then $x_{endo} = x_{endo}'$, $x_{exo} = x_{exo}'$ and $x = x'$.
4. The value of t is updated, $t' = t + \Delta t$, then $t = t'$.
5. Loop back to step 1.

The process starts at time $t = 0$, with $x_{endo} = x_{endo,eq}$ and $x_{exo} = x_{exo,eq}$.

3 Materials

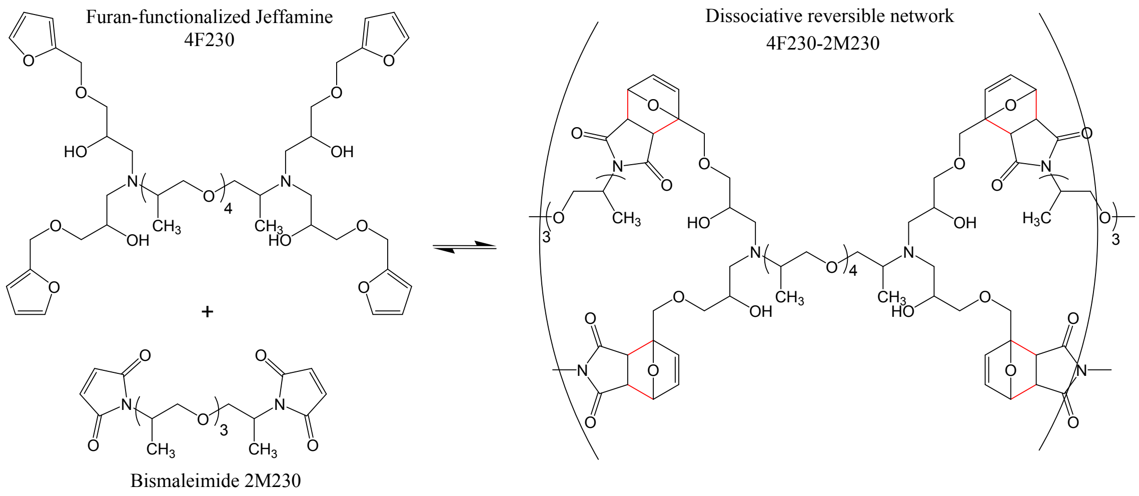

Scheme S - 1: Reversible network 4F230-2M230 formation and dissociation through Diels-Alder reaction. Reversible bonds are indicated in red.

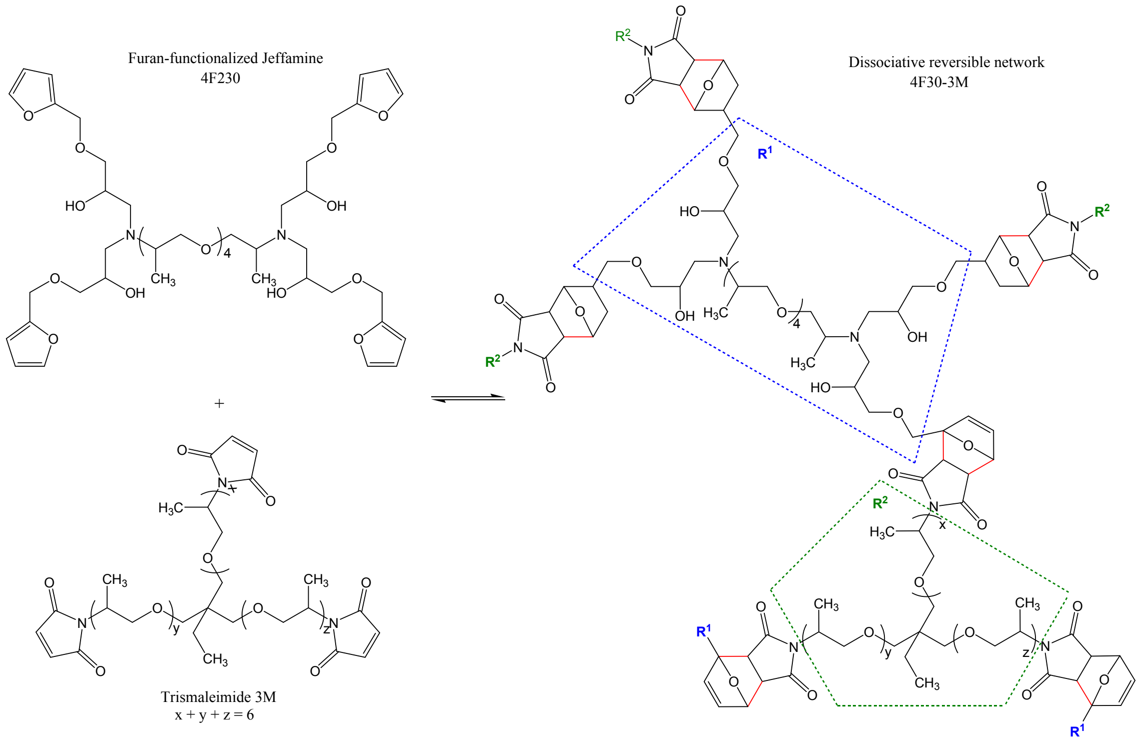

Scheme S - 2: Reversible network 4F230-3M formation and dissociation through Diels-Alder reaction. Reversible bonds are indicated in red.

4 Results

4.1 Analysis of model parameters

Analytical model

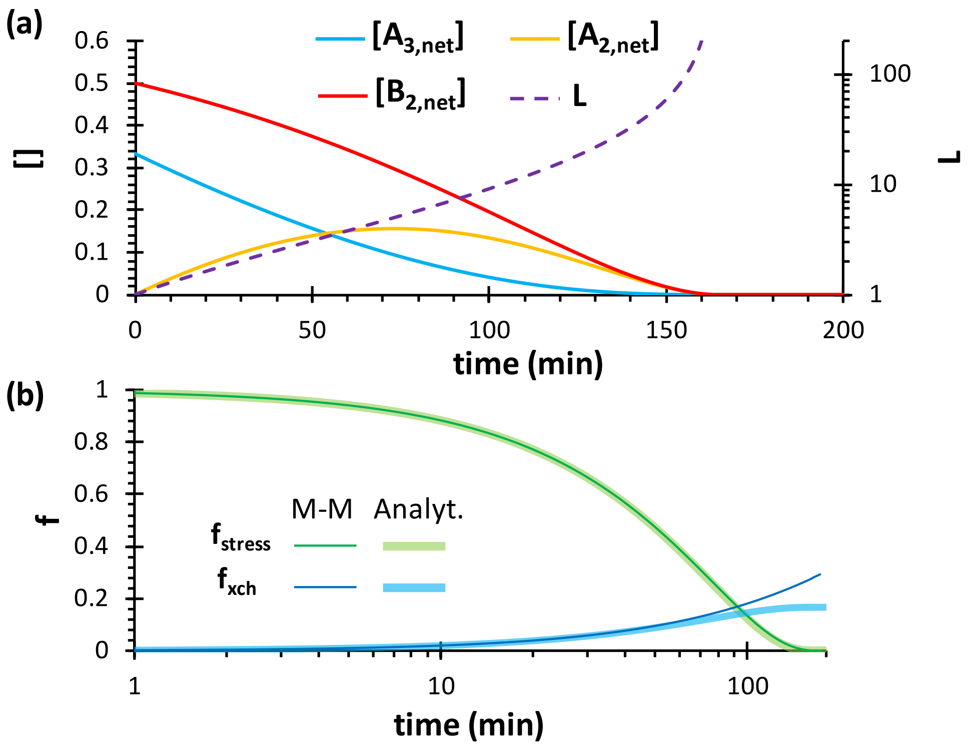

Figure S - 1: Outputs of the analytical model for $A_3 - B_2$ networks, showing (a) the evolution of the different species and average network strand length L , (b) the calculated relaxation stress f_{stress} and fraction of bond exchange events f_{xch}, in comparison with the M-M model with $f_{eff} = 1$. A total concentration of A and B groups equal to 1 (mol/kg) has been selected. A bond exchange rate constant of $k_{xch} = 0.002\ min^{-1}$ has been used for the calculations.

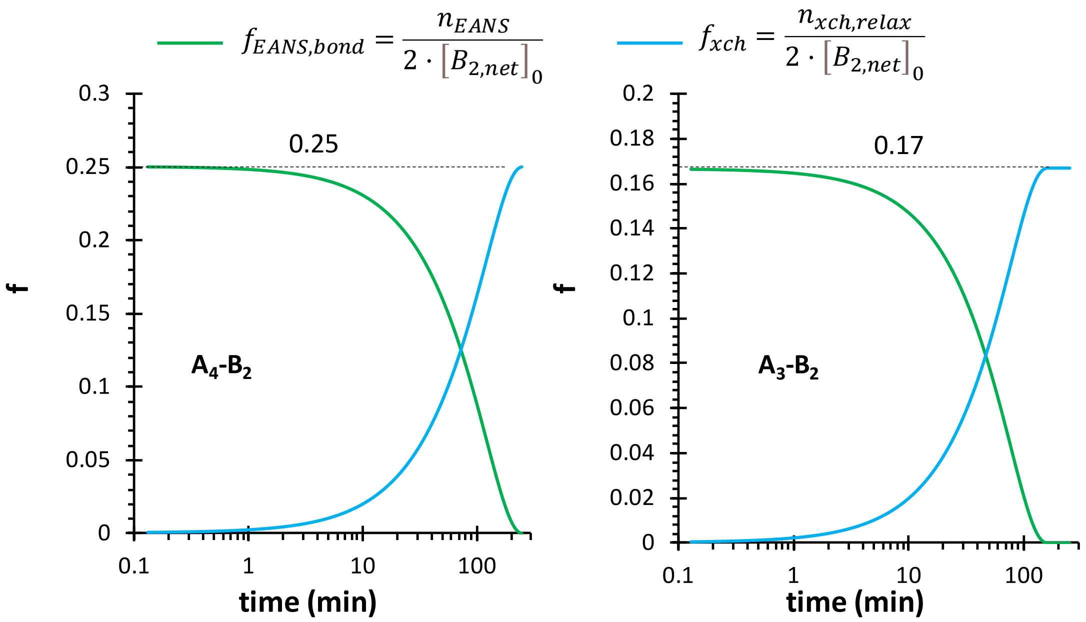

Figure S - 2: Comparison of the fraction of effective exchanged bonds and EANS with respect to the initial number of network bonds (f_{xch} and $f_{EANS,bond}$) respectively, for ideal fully crosslinked $A_4 - B_2$ and $A_3 - B_2$ networks. A total concentration of A and B groups equal to 1 (mol/kg) has been selected. A bond exchange rate constant of $k_{xch} = 0.002$ min^{-1} has been used for the calculations.

Effectiveness factor

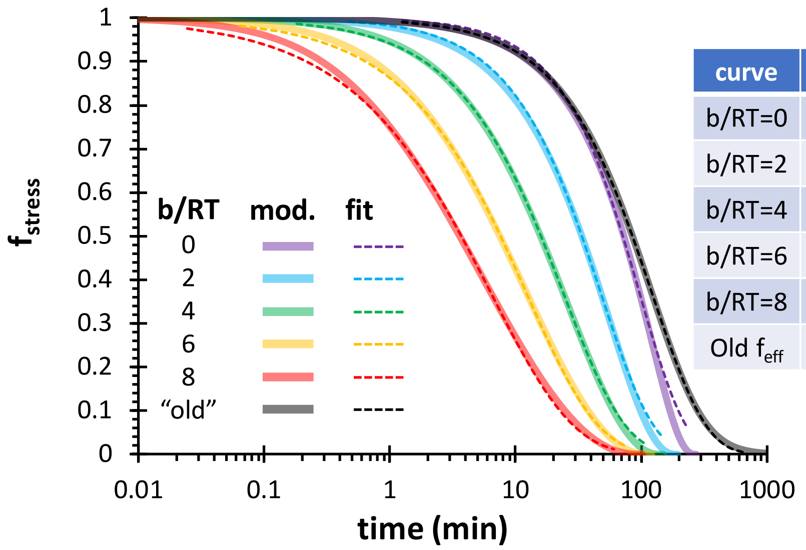

curve	τ	β	RSE
b/RT=0	97.50	1.180	0.0219
b/RT=2	47.97	1.039	0.0108
b/RT=4	24.15	0.882	0.0048
b/RT=6	12.30	0.761	0.0060
b/RT=8	6.43	0.664	0.0104
Old f_{eff}	122.16	1.010	0.0019

Figure S - 3: Analysis of the effect of the dimensionless parameter $b/R{\cdot}T$ on the stress relaxation kinetics of an $A_4 - B_2$ system using the M-M model, in comparison with the older definition of f_{eff}. A bond exchange constant k_{xch} equal to 0.002 min^{-1} was used for the calculations. Dashed lines represent the fitted curves to the stretched exponential

model, the results of the fit shown in the table. The side table shows the results of the fitting process including the residual standard error.

4.2 Analysis of experimental results

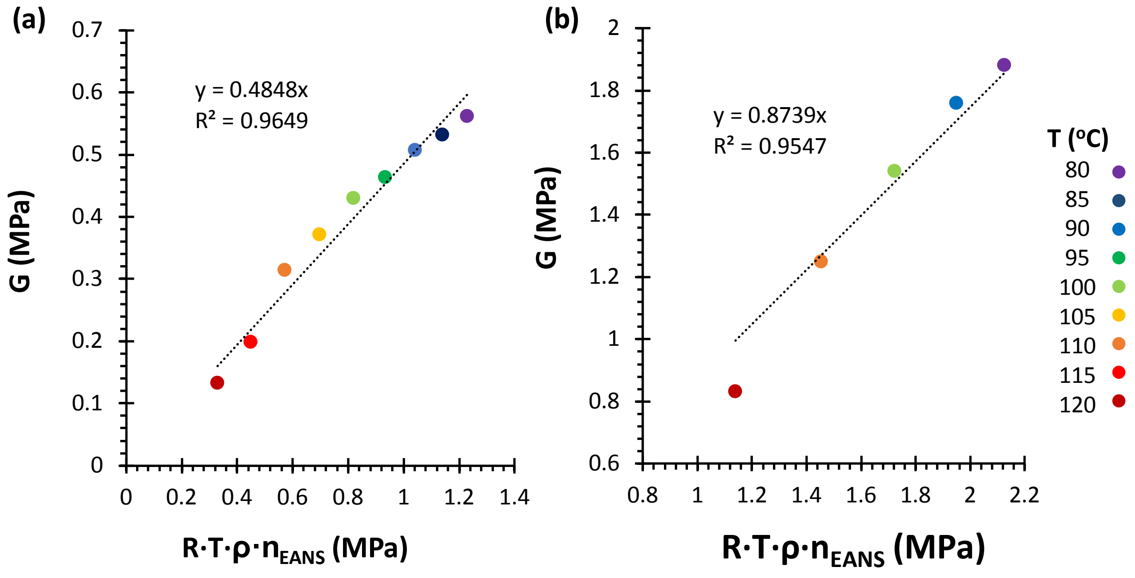

Figure S - 4: Comparison of equilibrium moduli G of 4F230-2M230 (a) and of 4F230-3M (b), both determined from the equilibrium relaxation moduli at the beginning of stress relaxation experiments, with $R{\cdot}T{\cdot}(n_{EANS}{\cdot}\rho)$, where n_{EANS} calculated using the model and ρ assumed to be 1100 kg·m^{-3}; the dashed line shows the linear regression according to the equation (9) in the manuscript. Rhelogical experiments were conducted using a gap of ca. 170 microns for 4F230-2M230 network and a gap of ca. 500 microns for 4F230-3M network.

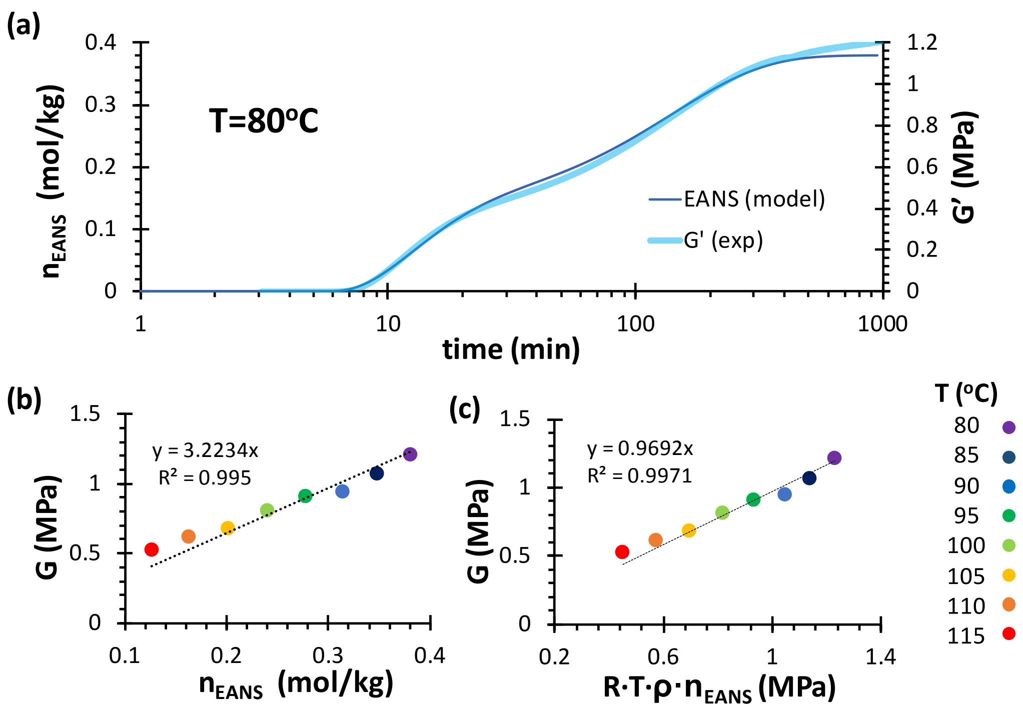

Figure S - 5: (a) Comparison of the evolution of *G*' during crosslinking of 4F230-2M230 at 80 °C with EANS calculated using the kinetic-structural model. (b) Comparison of equilibrium moduli *G* of 4F230-2M230M (determined from the equilibrium relaxation moduli at the beginning of stress relaxation experiments) and n_{EANS} calculated using the model; the dashed line shows the linear regression with intercept at the origin. (c) and comparison with $R{\cdot}T{\cdot}(n_{EANS}{\cdot}\rho)$, where n_{EANS} calculated using the model and ρ assumed to be 1100 kg·m^{-3}; the dashed line shows the linear regression according to the equation (9) in the manuscript. Rhelogical experiments were conducted using a gap of ca. 500 microns.

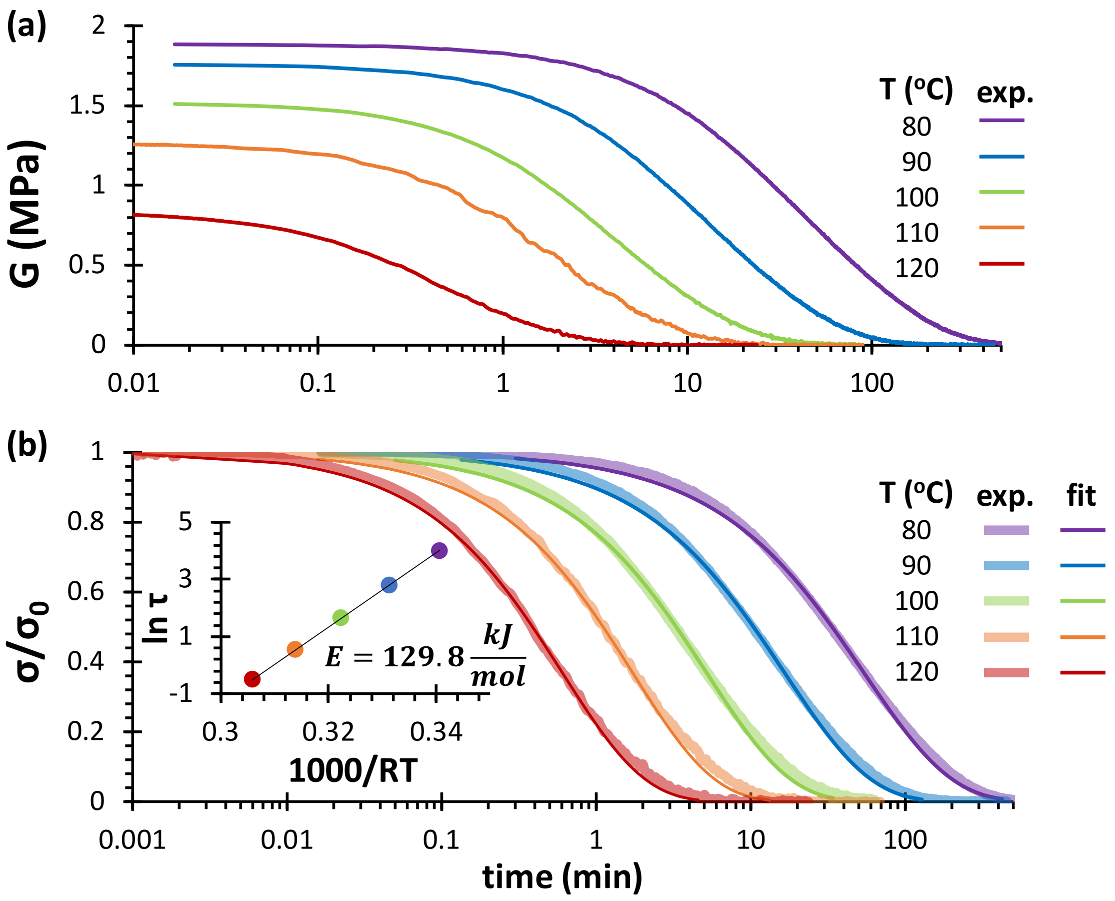

Figure S - 6: (a) Experimental stress relaxation curves of 4F230-3M networks (b) Normalized stress relaxation curves, fitting to stretched exponential model and determination of activation energy from relaxation times.

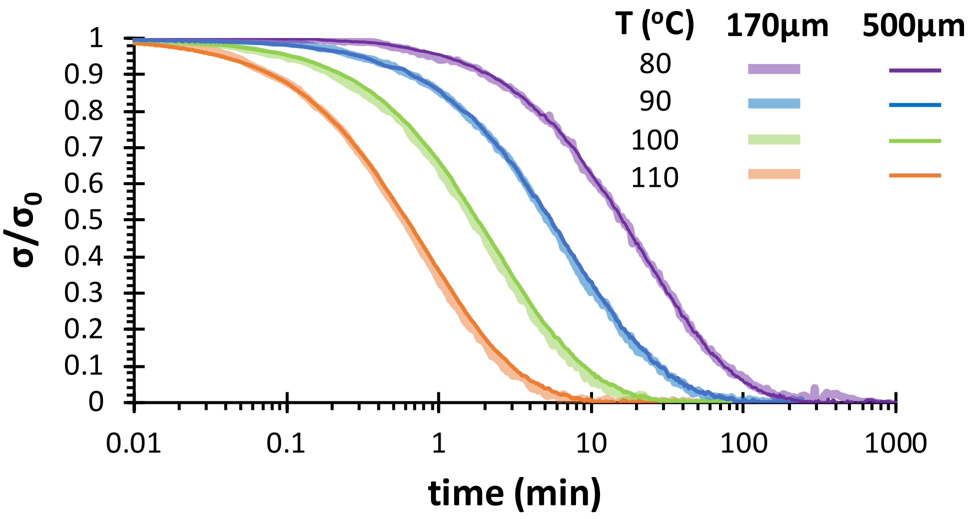

Figure S - 7: Comparison of normalized stress relaxation profiles of 4F230-2M230 network at different temperatures, determined from rheological experiments carried out with gaps of 170 and 500 microns.

Table S - 2: Results of the fitting of normalized experimental stress relaxation curves of 4F230-2M230 network to the stretched exponential model and analysis of the activation energy of the stress relaxation process.

	4F230-2M230			
T (°C)	$\tau\ (min)$	β	RSE	$\langle\tau\rangle\ (min)$
80	25.504	0.816	0.0104	28.494
85	14.444	0.802	0.0082	16.331
90	8.494	0.822	0.0134	9.444
95	4.927	0.809	0.0125	5.538
100	2.659	0.834	0.0096	2.929
105	1.558	0.841	0.0111	1.706
110	0.903	0.853	0.0102	0.980
115	0.546	0.871	0.0115	0.585
120	0.370	0.871	0.0135	0.397
$E_a = 124.72\ kJ/mol\quad (using\ \tau)$<br>$E_a = 126.26\ kJ/mol\quad (using\ \langle\tau\rangle)$				

Table S - 3: Results of the fitting of normalized experimental stress relaxation curves of 4F230-3M network to the stretched exponential model and analysis of the activation energy of the stress relaxation process. .

	4F230-3M			
T (°C)	$\tau\ (min)$	β	RSE	$\langle\tau\rangle\ (min)$
80	54.17	0.768	0.0115	63.28
90	16.62	0.785	0.0127	19.09
100	5.20	0.804	0.0135	5.87
110	1.73	0.825	0.0171	1.92
120	0.607	0.818	0.0129	0.677

$$E_a = 129.8\ kJ/mol \quad (using\ \tau)$$

$$E_a = 131.3\ kJ/mol \quad (using\ \langle\tau\rangle)$$

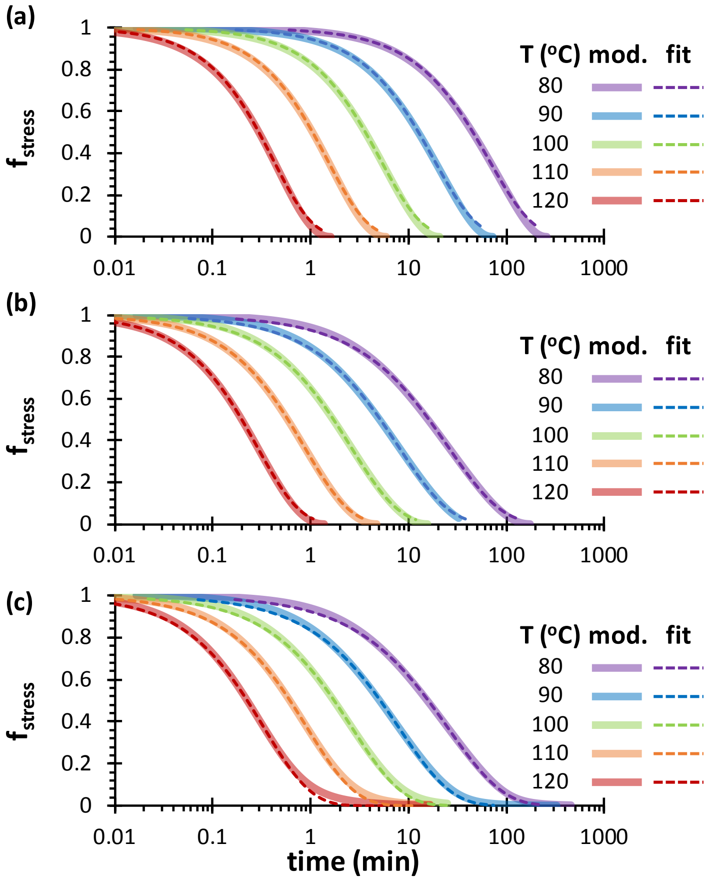

(a)

T (°C)	τ	β	RSE
80	66.35	0.9735	0.0112
90	18.27	0.9983	0.0093
100	5.186	1.0259	0.0085
110	1.485	1.056	0.0082
120	0.416	1.084	0.0093

(b)

T (°C)	τ	β	RSE
80	25.57	0.8003	0.007
90	7.98	0.8402	0.0057
100	2.593	0.8885	0.0043
110	0.867	0.9397	0.0033
120	0.286	0.9993	0.0045

(c)

T (°C)	τ	β	RSE
80	25.52	0.7769	0.0080
90	8.07	0.8169	0.0085
100	2.68	0.8530	0.0083
110	0.931	0.8875	0.0089
120	0.338	0.9071	0.0120

Figure S - 8: Results from the fit to the stretched-exponential model to the calculated stress relaxation curves of 4F230-2M230 networks. (a) M-M model with $b = 0$ ($f_{eff} = 1$), (b) M-M model with $b = 11.5\ kJ{\cdot}mol^{-1}$, (c) M-M model with $b = 12\ kJ{\cdot}mol^{-1}$, $a = 0.002$.

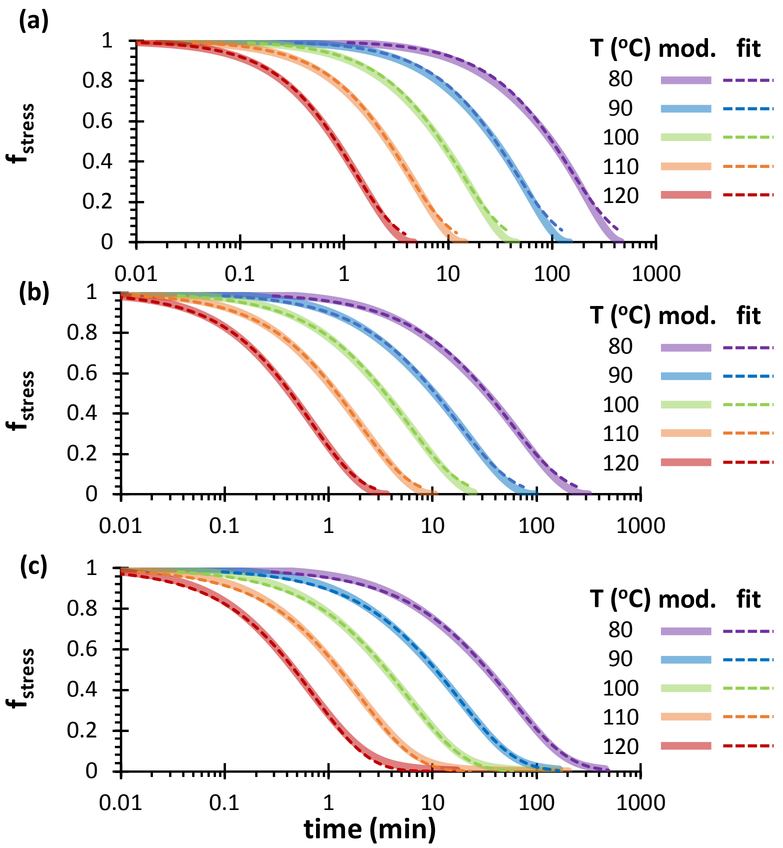

T (°C)	τ	β	RSE
80	147.18	0.9504	0.0227
90	42.15	0.9572	0.0198
100	12.56	0.9696	0.0162
110	3.85	0.9820	0.0132
120	1.21	1.0003	0.0107

T (°C)	τ	β	RSE
80	53.56	0.7863	0.0118
90	16.71	0.8025	0.0103
100	5.47	0.8243	0.0086
110	1.865	0.8506	0.0070
120	0.656	0.8852	0.0056

T (°C)	τ	β	RSE
80	54.68	0.7543	0.0065
90	17.25	0.7674	0.0058
100	5.74	0.7845	0.0062
110	2.00	0.8053	0.00795
120	0.731	0.8254	0.0104

Figure S - 9: Results from the fit to the stretched-exponential model to the calculated stress relaxation curves of 4F230-3M networks. (a) M-M model with $b = 0$ ($f_{eff} = 1$), (b) M-M model with $b = 11.5\ kJ{\cdot}mol^{-1}$, (c) M-M model with $b = 12\ kJ{\cdot}mol^{-1}$, $a = 0.004$.

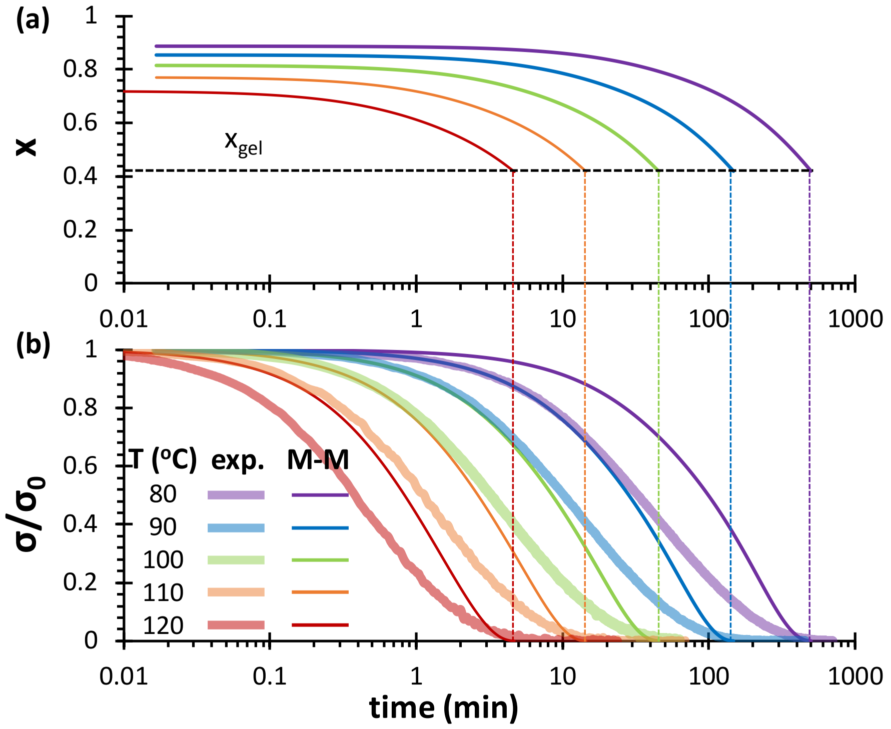

Figure S - 10: Simulation of the relaxation process of 4F230-3M network with the M-M model without considering the efficiency factor, that is, $f_{eff} = 1$. (a) evolution of conversion x and (b) normalized stress relaxation in comparison with experimental data.

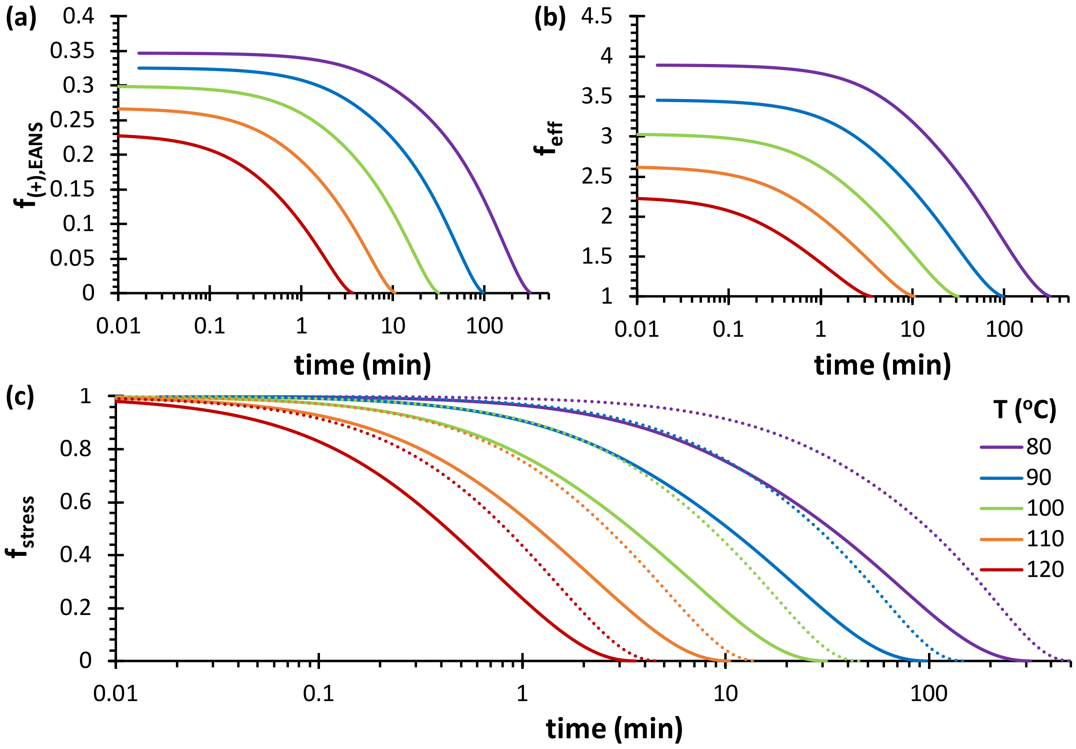

Figure S - 11: Detailed outputs of the modelling of the stress relaxation kinetics of 4F230-3M network using the M-M model with efficiency factor depending on b=11.5 kJ·mol-1, showing detailed evolution of (a)$f_{(+),EANS}$, (b) f_{eff} and (c) f_{stress}, in comparison with the M-M model with $f_{eff} = 1$ (dotted line).

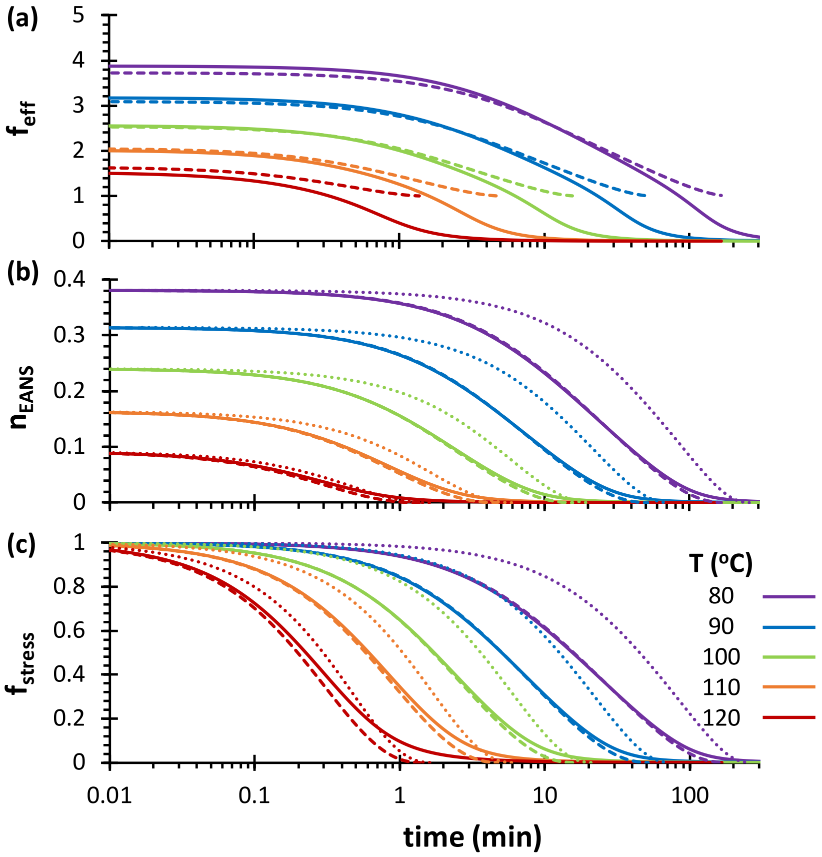

Figure S - 12: Comparison of the stress relaxation profiles of 4F230-2M230M network calculated using the M-M model without efficiency factor (dotted line), with f_{eff} depending on b=11.5 kJ·mol-1 (dashed line), and with f_{eff} depending on b=12 kJ·mol-1 and a=0.004 (solid lines). (a) f_{eff} (b) n_{EANS}, (c) f_{stress}.

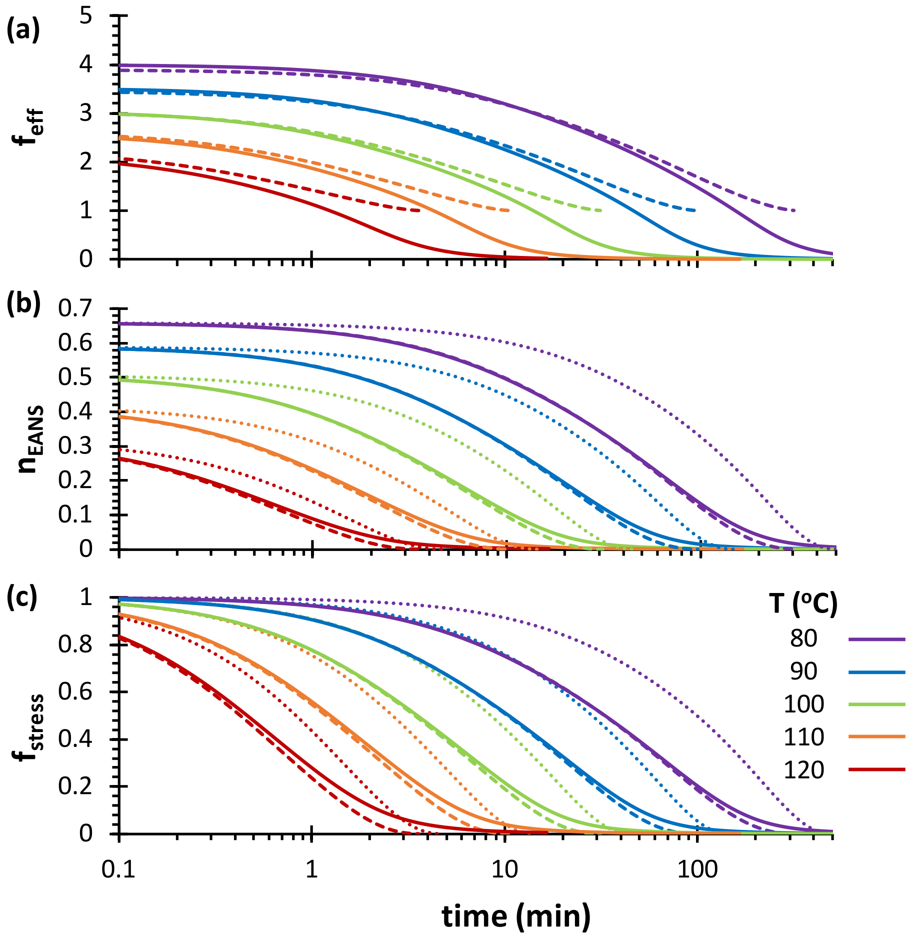

Figure S - 13: : Comparison of the stress relaxation profiles of 4F230-3M network calculated using the M-M model without efficiency factor (dotted line), with f_{eff} depending on b=11.5 kJ·mol^{-1} (dashed line), and with f_{eff} depending on b=12 kJ·mol^{-1} and a=0.004 (solid lines). (a) f_{eff} (b) n_{EANS}, (c) f_{stress}.

5 References

(1) Mangialetto, J.; Verhelle, R.; Van Assche, G.; Van den Brande, N.; Van Mele, B. Time-Temperature-Transformation, Temperature-Conversion-Transformation, and Continuous-Heating-Transformation Diagrams of Reversible Covalent Polymer Networks. *Macromolecules* **2021**, *54* (1), 412–425. https://doi.org/10.1021/acs.macromol.0c02491.

(2) Cuvellier, A.; Verhelle, R.; Brancart, J.; Vanderborght, B.; Van Assche, G.; Rahier, H. The Influence of Stereochemistry on the Reactivity of the Diels–Alder Cycloaddition and the Implications for Reversible Network Polymerization. *Polym. Chem.* **2019**, *10* (4), 473–485. https://doi.org/10.1039/C8PY01216D.

(3) Miller, D. R.; Valles, E. M.; Macosko, C. W. Calculation of Molecular Parameters for Stepwise Polyfunctional Polymerization. *Polym. Eng. Sci.* **1979**, *19* (4), 272–283.

(4) Pascault, J.-P. P.; Sautereau, H.; Verdu, J.; Williams, R. J. J. *Thermosetting Polymers*; Marcel Dekker: New York [etc.] :, 2002.

(5) Konuray, O.; Fernández-Francos, X.; Ramis, X. Structural Design of CANs with Fine-Tunable Relaxation Properties: A Theoretical Framework Based on Network Structure and Kinetics Modeling. *Macromolecules* **2023**, *56* (13), 4855–4873. https://doi.org/10.1021/acs.macromol.3c00482.
